# Functional Characterization of Genes Coding for Novel *β*-D-Glucosidases Involved in the Initial Step of Secoiridoid Glucosides Catabolism in *Centaurium erythraea* Rafn

**DOI:** 10.3389/fpls.2022.914138

**Published:** 2022-06-23

**Authors:** Jelena Božunović, Milica Milutinović, Neda Aničić, Marijana Skorić, Dragana Matekalo, Suzana Živković, Milan Dragićević, Biljana Filipović, Tijana Banjanac, Luka Petrović, Danijela Mišić

**Affiliations:** Department of Plant Physiology, Institute for Biological Research “Siniša Stanković”- National Institute of the Republic of Serbia, University of Belgrade, Belgrade, Serbia

**Keywords:** beta glucosidase, secoiridoid glucosides, functional characterization, agroinfiltration, *Centaurium erythraea*

## Abstract

Secoiridoid glucosides (SGs) are monoterpenoids derived from the iridoid cyclopentane-C-pyran skeleton with *β*-D glucose linked at C1 position. Coordinated metabolic processes, such as biosynthesis and catabolism of SGs, ensure constitutive presence of these bitter tasting compounds in plant tissues, which plays a decisive role in the defense against pathogens and herbivores. These compounds are susceptible to hydrolysis mediated by enzymes *β*-glucosidases, and the resulting aglycones are subsequently directed toward different metabolic pathways in plants. Function of two *β*-D-glucosidases (named *Ce*BGlu1 and *Ce*BGlu2) from centaury (*Centaurium erythraea* Rafn; fam. Gentianaceae), belonging to the glycoside hydrolase 1 (GH1) family, was confirmed using *in vitro* assays with recombinant proteins, following their heterologous expression in *E. coli* and His-tag affinity purification. Although they show slightly differential substrate preference, both isoforms display high specificity toward SGs and the organ-specific distribution of transcripts was positively correlated with the content of SGs in diploid and tetraploid *C. erythraea* plants. Transient overexpression of *Ce*BGlu1 and *Ce*BGlu2 in *C. erythraea* leaves induced changes in metabolite profiles. The effectiveness of transgene overexpression has been altered by plant ploidy. UHPLC/DAD/(±)HESI − MS^2^ profiling of leaves of diploid and tetraploid *C. erythraea* genotypes revealed that the amounts of major SGs; sweroside, swertiamarin, and gentiopicrin was decreased in agroinfiltrated leaves, especially when *Ce*BGlu1 and *Ce*BGlu2 were co-expressed with transgene silencing suppressor p19. The work demonstrates that *in planta* metabolic engineering adopting transient overexpression of *Ce*BGlu1 and *Ce*BGlu2 is a suitable tool for the modulation of SGs content and glucosides/aglycones ratio, which might have substantial effects on overall phytochemistry of *C. erythraea*.

## Introduction

**FIGURE 1:**
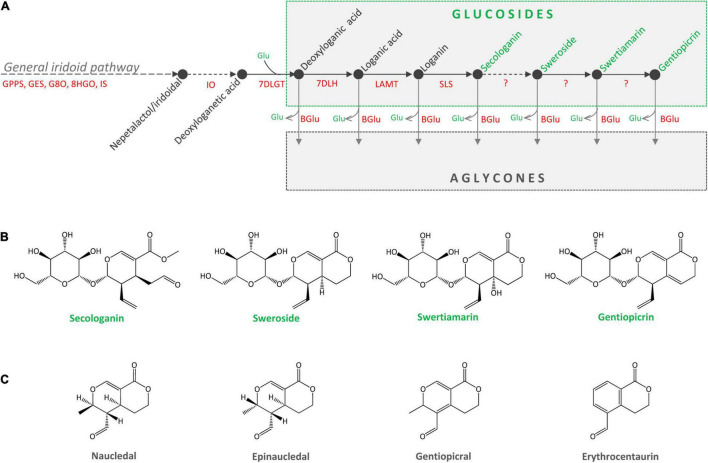
**(A)** Proposed metabolic pathway of secoiridoid glucosides in *Centaurium erythraea*. Unknown enzymes of the biosynthetic pathway are marked with the question mark. GPPS, geranyl diphosphate synthase; GES, geraniol synthase; G8O, geraniol-8-oxidase; 8HGO, 8-hydrohygeraniol oxidoreductase; IS, iridoid synthase; IO, iridoid oxidase; 7DLGT, 7-deoxyloganetic acid glucosyltransferase; 7DLH, 7-deoxyloganic acid hydrolase; LAMT, loganic acid O-methyltransferase; SLS, secologanin synthase; BGlu, beta glucosidase. **(B)** Major secoiridoid glucosides of *C. erythraea*, secologanin, sweroside, swertiamarin, and gentiopicrin. **(C)** Aglycones of sweroside (naucledal and epinaucledal), swertiamarin and gentiopicrin (gentiopicral and erythrocentaurin).

Secoiridoid glucosides (SGs) are a group of plant-derived natural compounds widely present in species belonging to orders Gentianales, Dipsacales, Cornales, and Lamiales (family Oleaceae) (Ghisalberti, 1998; Jensen et al., 2002). Species of the genus *Centaurium* Hill (fam. Gentianaceae) are a rich source of SGs, among which sweroside (SW), swertiamarin (SWM), and gentiopicrin (GP) predominate. Leaves are the major site of SGs biosynthesis and accumulation in *C. erythraea* (Matekalo et al., 2018). Secoiridoids are named after secologanin (SEC), derived after the cleavage of the cyclopentane ring of iridoids (between C-7 and C-8). Secologanin is considered the common precursor of all secoiridoids in plant sources (Jensen and Schripsema, 2002), but is also the building block for monoterpene indole alkaloids (MIAs). *Centaurium* species are presumed to share a part of the secoiridoid biosynthetic pathway up to SEC with MIAs-producing *Catharanthus roseus*. The biosynthetic pathway of SEC starts with the synthesis of geranyl diphosphate (GPP), which is converted to geraniol and continues via a number of intermediates such are 8-hydroxygeraniol, 8-oxogeraniol, nepetalactol, iridotrial, 7-deoxyloganetic acid, 7-deoxyloganic acid, loganic acid and loganin (Figure 1A). The biosynthetic pathway involves a series of reactions, catalyzed by enzymes geranyl diphosphate synthase (GPPS), geraniol-8-oxidase (G8O), 8-hidroxygenaniol oxidoreductase (8HGO), iridoid synthase (IS), iridoid oxidase (IO), 7-deoxyloganetic acid glucosyltransferase (7DLGT), 7-deoxyloganic acid hydrolase (7DLH), loganic acid O-methyltransferase (LAMT), and secologanin synthase (SLS). In *C. roseus*, SEC and tryptamine are precursors of monoterpenoid indole alkaloids (MIAs) such as strictosidine, while in *C. erythraea* SEC is metabolized into secoiridoid glucosides SW, SWM and GP, as well as their derivatives. Enzymes responsible for the conversion of SEC to SW, and further to SWM and GP are not elucidated yet (Figure 1A).

The content of SGs in *C. erythraea* tissues is a net result of the two dynamic metabolic processes, their biosynthesis and their catabolism (Figure 1A). Catabolism of SGs starts with their deglycosylation catalyzed by *β*-glucosidases. Cleavage of the glucose (Glu) residue from SW, SWM, and GP results in the formation of aglycones (Figures 1A,B), which are further metabolized in plant tissues through isomerization, reduction and oxidation reactions. Gentiopicral and erythrocentaurin are the common products of SWM and GP hydrolysis (Ishiguro et al., 1983; Zeng et al., 2013; Božunović et al., 2018), while SW is metabolized into naucledal and epinaucledal (Purdy and McLean, 1977; El-Sedawy et al., 1990) (Figure 1C). In order to better understand the catabolism of SGs in *C. erythraea*, we focused our attention on *β*-glucosidases (*β*-D-glucoside glucohydrolases, E.C. 3.2.1.21), categorized into the glycoside hydrolase family 1 (GH1), the largest GH family in plants. GH1 *β*-glucosidases are enzymes that hydrolyze glycosidic bonds to release non-reducing terminal glucosyl residues from various compounds – benzoxazinoid, cyanogenic, iridoid and phenolic glucosides – as well as glucosinolates (Morant et al., 2008a). Genes coding for *β*-glucosidase (*β*-Glu) enzymes have been previously characterized in some members of the order Gentianales, including *C. roseus* and *Rauvolfia serpentina* (Geerlings et al., 2000; Warzecha et al., 2000; Barleben et al., 2007). Isolation and heterologous expression of *C. roseus β*-glucosidase with high specificity toward strictosidine which directly derives from SEC, provided valuable information on the function of this *β*-glucosidase involved in metabolic pathway of indole alkaloids (Geerlings et al., 2000). The aim of the present study was to isolate and functionally characterize *C. erythraea β*-glucosidase with high substrate specificity toward SGs using *in vitro* and *in planta* assays. Our presumption was that the action of *C. erythraea β*-glucosidase with high specificity toward SGs is essential for the catabolism of these compounds, and is indirectly related to defense against herbivores and pathogens. Secoiridoids in the form of glucosides possess remarkable antimicrobial effects (Božunović et al., 2018), and their hydrolysis/deglycosylation mediated by *β*-D-glucosidases release biologically active aglycones which provide more efficient antioxidant protection (Božunović et al., 2018). Moreover, during hydrolysis of SGs, glucose is also released, which may serve as an alternative source of energy under stress conditions.

## Materials and Methods

### Plant Material

Plants used in experiments *in vitro* were obtained as previously described by Filipović et al. (2019). Briefly, mother stock shoot cultures of *C. erythraea* diploids and tetraploids originating from seeds collected at the locality Tjentište (Sutjeska National Park, Bosnia and Herzegovina), in 2007 and 2016, respectively, were maintained *in vitro* on half–strength MS medium (^1^/_2_ MS, Murashige and Skoog, 1962) in 370 ml glass jars. Root cultures, established from root segments of 3-month-old diploid and tetraploid plants, were grown on solid 1/2 MS medium in Petri dishes. Spontaneously regenerated shoots, formed on root explants, were further transferred on fresh 1/2 MS medium in 370 ml glass jars for rooting. After 10 weeks of culturing, shoots and roots of the obtained regenerated plants were harvested and used in experiments to determine the expression patterns of *Ce*BGlu candidates and their activities. All *in vitro* cultures were maintained at a temperature of 25 ± 2°C under fluorescent light of 47 μmol s^–1^ m^2^ and a 16 h/8 h light/dark photoperiod.

Agroinfiltration experiments were performed under greenhouse conditions. *C. erythraea* seedlings were established in greenhouse, in pots filled with Floradur B seed substrate for multiplication (Floragard Vertriebs-GmbH, Oldenburg, Germany). Two-month-old seedlings were individually transferred into pots with Floragard growth medium (Floragard Vertriebs-GmbH, Oldenburg, Germany), and grown under greenhouse conditions at 50–85% humidity. Five-month-old diploid and tetraploid plants, displaying rosette phenotype, were used in agroinfiltration experiments. Seeds of tetraploid *C. erythraea*, collected in 2006, were obtained from Ecological-Botanical Garden of the University of Bayreuth (Germany). Diploid individuals were of the same origin as those used in *in vitro* experiments. All the *C. erythraea* accessions used in the present study are deposited within the seed collection at the Department of Plant Physiology, Institute for Biological Research “Siniša Stanković” – National Institute of the Republic of Serbia, University of Belgrade (Serbia).

### RNA Isolation and cDNA Synthesis

Total RNA from approximately 150 mg of *C. erythraea* shoots and roots was isolated using modified CTAB method (Gasic et al., 2004). Isolated RNA was quantified using Qubit 3.0 Fluorometer (Thermo Fisher Scientific, United States), and its integrity was confirmed using gel electrophoresis. Prior to RT-PCR, isolated RNA was treated with DNAse I (Thermo Fisher Scientific, United States) to deplete contaminating genomic DNA. First strand cDNA was synthesized from 300 μg RNA using the RevertAid Premium First Strand cDNA Synthesis Kit (Thermo Fisher Scientific, United States).

### Isolation and Cloning of *β*-Glucosidase Candidate Genes

*Centaurium erythraea* leaf transcriptome databases (Malkov and Simonović, 2011; Ćuković et al., 2020) was searched for homologs of *Catharanthus roseus* strictosidine *β*-glucosidase (Geerlings et al., 2000). After selecting the most promising candidate, gene specific primers were designed using Primer3Plus software^1^ in order to amplify the full length of the *Ce*BGlu coding sequence. A list of primers used for cloning and expression analysis is given in Supplementary Table 1.

The full length amplification of the candidate gene was performed using Q5 Hot Start High-Fidelity DNA Polymerase (New England Biolabs, United States) and gene specific forward and reverse primers (Supplementary Table 1) following cycling conditions: one cycle of 98°C for 3 min, 35 cycles of 98°C for 30 s, 62°C for 2 min, and 72°C for 5 min followed by a final extension of 72°C for 10 min in a thermal cycler (Eppendorf, Austria). The amplicon was separated by 1% agarose gel electrophoresis and then purified by GeneJET Gel Extraction Kit (Thermo Fisher Scientific, United States) according to manufacturer’s instructions. The purified amplicon was cloned into pTZ57R/T cloning vector using T/A PCR product cloning kit (Thermo Fisher Scientific, United States). In summary, 20 μl of purified PCR product was mixed with 3 μl of pTZ57R plasmid vector (600 ng), 6 μl of 5× ligation buffer, 1 μl of T4 DNA Ligase (5 U μl^–1^), in 1.5 ml microtube. A total of 30 μl reaction mixture was incubated for 1 h at room temperature to let the ligation reaction take place.

For transformation, 5 μl of the ligation product was added into 100 μl of Mach1 *E. coli* competent cells, incubated on ice for 20 min and then heat-shocked for 45 s in water bath at 42°C. The mixtures were immediately placed on ice and subsequently cultivated with 250 μl liquid Luria-Bertani (LB) media followed by 1 h incubation at 37°C. The transformed cells were cultivated on LB agar plate containing ampicillin (100 μg ml^–1^), which was followed by overnight incubation at 37°C. Positive transformants were verified by colony PCR with *Ce*BGlu gene-specific primers (Supplementary Table 1). Cells harboring the recombinant plasmid were cultured in ampicillin containing liquid LB medium overnight at 37°C in a shaker incubator at 220 rpm. GeneJET Plasmid Miniprep Kit (Thermo Fisher Scientific, United States) was used to purify plasmids from 2 ml of Mach1 *E. coli* overnight culture according to the manufacturer’s instructions. Recombinant plasmids were confirmed by restriction digestion using *Xho*I and *Kpn*I restriction enzymes (Thermo Fisher Scientific, United States). Subsequent sequencing has confirmed the isolation of two highly similar gene variants named *CeBGlu1* and *CeBGlu2*.

### Organ-Specific Profiling of *CeBGlu* Expression

For qRT-PCR, due to the very high similarity between *CeBGlu1* and *CeBGlu2* sequences, it was possible to design only one pair of primers common to both isoforms (Supplementary Table 1). For *CeBGlu* expression analysis, SYBR Green I (Maxima SYBR Green/ROX Kit, Thermo Scientific, United States) was used. Amplification was conducted in Light cycler QuantStudio 3 (Thermo Fisher Scientific, United States), according to the manufacturer’s instructions. General thermocycler conditions were 95°C for 10 min; 40 cycles of 95°C for 15 s; 60°C for 30 s; 72°C for 30 s and final extension at 72°C for 10 min. Expression levels of targeted genes were calculated according to the 2^–ΔΔCt^ method (Livak and Schmittgen, 2001). *EF1* gene expression was used as endogenous control to normalize all data (primer sequences are presented within Supplementary Table 1). Presented results are obtained from three biological replicates.

### Organ-Specific Profiling of Total Hydrolytic Activity Against Secoiridoid Glycosides

Shoots and roots of diploid and tetraploid *C. erythraea* plants were ground to a fine powder using liquid nitrogen, and proteins were isolated in 100 mM potassium-phosphate buffer (pH 6.5) supplemented with phenylmethylsulfonyl fluoride (PMSF) and 5 mM ascorbate. Protein content was determined according to Bradford (1976) using bovine serum albumin as a standard.

Activity of protein extracts to reduce the content of secoiridoid glycosides was evaluated using standards of SW, SWM and GP as substrates. The reduction in quantity of the mentioned substrates is the net result of combined enzymatic activities present in the extract including glucosyl hydrolases which produce the respective aglycones and unknown biosynthetic enzymes involved in transformations of secoiridoids (Figure 1A).

All reference compounds were diluted in dH2O (w:v = 1:1) and kept as a stock solutions. Aliquots of 30 μl were vacuum evaporated and diluted in 300 μl 50 mM citrate phosphate buffer pH 5.5 containing 10 μg of protein extracts. Reactions were incubated for 24 h at 37°C. Subsequently, 700 μl of methanol was added, and samples were centrifuged at 10,000 *g* for 10 min. Supernatants were filtered through 0.2 μm cellulose filters and subsequently analyzed for the content of SW, SWM and GP.

### Phylogenetic Analysis

For phylogenetic tree creation, multiple sequence alignment-s were generated using the Muscle algorithm. A neighbor-joining tree was constructed using MEGA X, Version 10.2.6 (Kumar et al., 2018). Cluster stability was estimated with 1,000 bootstrap replicates. The evolutionary distances were computed using the Poisson correction method. All ambiguous positions were removed for each sequence pair (pairwise deletion option). There were a total of 766 positions in the final dataset. The data were converted into Newick format and transferred to Dendroscope (Huson and Scornavacca, 2012) for creating the final phylogenetic tree. Refer to Supplementary Table 2 for a list of plant protein sequences used for phylogenetic analysis and their corresponding accession numbers, including those of *Ce*BGlu1 and *Ce*BGlu2 functionally characterized within the present study.

### Sequence Analysis, 3D Modeling and Ligand Docking

Multiple sequence alignments were generated with the DECIPHER R package (Wright, 2015) using 5 iterations and 5 refinements. Subcellular localization based on primary sequence was estimated using Light attention (Stärk et al., 2021).

Tertiary protein structure was estimated using AlphaFold2.1 (Jumper et al., 2021) queried via UCSF ChimeraX 1.4rc (Pettersen et al., 2021). The generated structures were assessed using MolProbity 4.4 (Williams et al., 2018) via SWISS-MODEL Workspace (Waterhouse et al., 2018). Obtained models were compared to each other and with experimental PDB structures using pairwise structure alignment^2^ and the jFATCAT rigid model (Li et al., 2020), as well as using ChimeraX after superposition using the matchmaker command with default parameters (Pettersen et al., 2021). Protonation state for the predicted structures was estimated using PlayMolecule ProteinPrepare (Martínez-Rosell et al., 2017). Ligand docking was performed using AlphaFold predicted protein structure of *Ce*BGlu1. Ligand preparation was performed starting from tridimensional sdf files obtained from PubChem for SWM (PubChem id: 442435), SW (PubChem id: 161036) and GP (PubChem id: 88708). Conformers for these compounds were generated using the ETKDG version 3 method with imposing small ring torsion angle preferences (Wang et al., 2020) using RDKit 2021.09.5 (Landrum et al., 2022). A 100 conformers were generated per compound and subsequently filtered using an RMSD threshold of 0.5 Å so that only those conformations that are at least 0.5 Å RMSD away from all retained are kept. The geometry of the resulting conformers was optimized using Merck molecular force field (MMFF94s) as implemented in RDKit 2021.09.5 (Landrum et al., 2022). The resulting ligand conformations were docked into the predicted enzyme structure using AutoDock Vina 1.2.3 (Eberhardt et al., 2021). The docking box was defined based on the coordinates of the malo secologanin ligand in the experimental structure of the raucaffricine *β*-D glucosidase from *Rauvolfia serpentina* (PDB: 3U5Y, Xia et al., 2012) after superposition of the structure to the AlphaFold model of *Ce*BGlu1. The docking box center was defined as *x* = −1.964, *y* = 0.374, *z* = −6.148, while the size of the box was 18, 18.75, and 21 Å in each direction, respectively. The docking was performed using autogrid4 (Morris et al., 2009) precalculated affinity maps, with autodock4 scoring function. Flexible docking was performed where the Glu476 side chain was allowed to change conformations. The exhaustiveness of the algorithm was set to 64. The highest estimated affinity poses, as reported by AutoDock Vina, were inspected and compared to the position of secologanin in 3U5Y, and the best pose based on these two criteria for each ligand was analyzed using UCSF ChimeraX 1.4rc (Pettersen et al., 2021) and protein–ligand interaction profiler (PLIP, Adasme et al., 2021).

### Accession Numbers

Sequence data from this article can be found in the NCBI database under the following accession numbers: ON060690 (*Ce*BGlu1); ON060691 (*Ce*BGlu2).

### Heterologous Expression and Purification of 6His-Tagged Proteins

**FIGURE 2:**
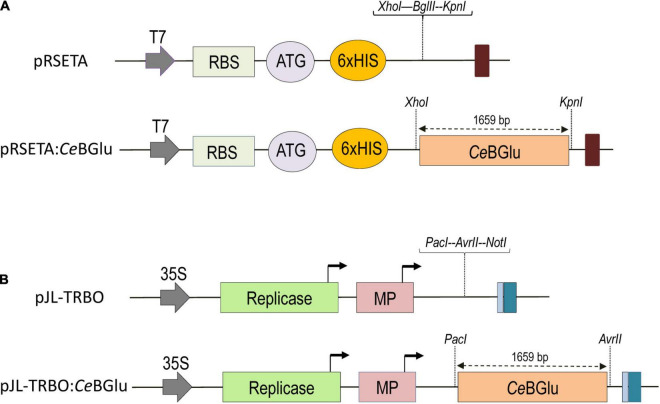
Maps of vectors used in this study, pRSETA for the expression in *E.coli* **(A)** and pJL-TRBO for overexpression in *C. erythraea* **(B)**. CeBGlu, *β*-D-glucosidase from *C. erythraea*, was inserted as a XhoI-KpnI fragment into pRSETA expression vector **(A)**, or as PacI-AvrII fragment into pJL-TRBO **(B)** to produce pRSETA:CeBGlu and pJL-TRBO:CeBGlu constructs, respectively. The T-DNA regions of binary plasmids are presented. Block arrow, CaMV duplicated 35S promoter and T7 promoter. Dark blue box, CaMV polyA signal sequence/terminator. Light blue box, Ribozyme. Bent arrows, Subgenomic promoters. ORFs are represented by colored boxes and their identities are labeled in boxes. Replicase, TMV 126K/183K ORF; MP, movement protein; RBS, ribosome binding site; Colored oval boxes present start codon (ATG) and polyhistidine-tag sequence (6xHIS).

For heterologous expression, *CeBGlu1* and *CeBGlu2* were cloned into bacterial expression vector pRSETA, yielding the final plasmids pRSETA:*CeBGlu1* and pRSETA:*CeBGlu2* (Figure 2A). Amplification of the gene sequences by PCR was performed using Phusion high-fidelity DNA polymerase (Thermo Fisher Scientific, United States) and a pair of *Ce*BGlu1/*Ce*BGlu2 gene specific primers with added *Xho*I/*Kpn*I restriction sites (Supplementary Table 1). The constructs were heat-shock transformed, as described above, into Mach1 *E. coli* competent cells which were further cultured on LB medium containing ampicillin. Polymerase chain reaction using gene specific primers, *Xho*I/*Kpn*I double restriction digestion and sequencing were applied to verify the constructs containing transgenes of interest.

pRSETA vector contains a N-terminal polyhistidine (6×His) tag, which can be used as an affinity ligand for protein purification. The pRSETA:*Ce*BGlu1 and pRSETA:*Ce*BGlu2 recombinant constructs were used for heat-shock transformation of BL21 (DE3) CodonPlus-RIL cells (Stratagene, United States). Cultures were grown overnight in 20 ml 2 × Yeast Extract Tryptone (2 × YT) media broth supplemented with ampicillin (100 μg ml^–1^), 50 μg ml^–1^ kanamycin and 17 μg ml^–1^ chloramphenicol at 37°C with shaking at 220 rpm. On the next day 200 ml culture was initiated by inoculating above mentioned overnight cultures, with the initial OD600 set to 0.1. Cultures were incubated at 37°C with shaking at 230 rpm. After reaching an OD600 of 0.4–0.5, protein expression was induced using 0.1 mM isopropyl-*β*-D-thiogalactopyranoside (IPTG). Subsequently, incubation continued for 4 h at 21°C. Following centrifugation for 10 min at 5,000 *g* and removal of supernatant, the cell pellets were harvested. All of the purification steps were performed at 4°C. For each purification batch, Ni-NTA agarose (Qiagen, United States) beads were equilibrated with equal volumes of lysis buffer (50 mM NaH_2_PO_4_, 300 mM NaCl, 10 mM imidazole pH 8.0). Bacterial cells were collected by centrifugation and re-suspended in 500 μl of lysis buffer. Following lysozyme (Sigma Aldrich, Germany) addition (1 mg ml^–1^ final concentration), samples were incubated for 30 min on ice. After 10 min of centrifugation (12,000 *g* at 4°C) supernatants were loaded into columns containing Ni–NTA resin and incubated at 4°C using FALC F205 rotary tube mixer (Falc Instruments, Treviglio, Italy). After 1 h, samples were spun at 12,000 g for 5 min at 4°C. Supernatant was discarded and resin was rinsed for 3 times using washing buffer (50 mM NaH_2_PO_4_, 300 mM NaCl, 50 mM imidazole, pH 8.0). The recombinant protein was eluted with increased imidazole concentration in elution buffer (50 mM NaH_2_PO_4_, 300 mM NaCl, 250 mM imidazole, pH 10.2).

Eluted fractions were analyzed using 5–10% SDS-PAGE using Mini-PROTEAN II Electrophoresis Cell (Bio-Rad, United States) followed by Coomassie blue staining and immunoblotting. Proteins were electro-transferred to PVDF membrane (Amersham Biosciences, Germany), using Mini Trans-Blot Module electric transfer system (Bio-Rad, United States). Transfer on the membrane was performed at 4°C for 90 min at a constant voltage of 60 V. Membrane was blocked with 10% (w/v) non-fat dry milk (NFDM; Nestle, United States) in phosphate-buffered saline containing 0.05% Tween-20 overnight at 4°C. The presence of 6×His labeled proteins was confirmed using His-probe antibody in 1:100 dilution (Acc. No. sc-53073, Santa Cruz Biotechnology, United States), which was followed by incubation with goat anti-mouse IgG-HPR (1:5,000, Agrisera Antibodies, Sweden). The bound antibodies were visualized by enhanced chemiluminescence (ECL). The membrane was incubated for 5 min at room temperature using ECL solution containing 100 mM Tris–HCl pH 8.5, 0.2 mM *p*-coumaric acid, 1.25 mM 3-aminophthalhydrazide and 1.7 μl of 30% H_2_O_2_. Detection was performed by exposure to radiographic film (Medical X-ray Green/MXG Film, Carestream Health, United States) for 10 min.

### Confirmation of *Ce*BGlu1 or *Ce*BGlu2 Hydrolytic Activity Using *in vitro* Enzymatic Assays

Beta-glucosidase activity of *Ce*BGlu1 and *Ce*BGlu2 was determined in a reaction with 20 μg of recombinant proteins and 10 mM 4-nitrophenyl *β*-D-glucopyranoside (*p*NPG) as a substrate, in 50 mM citric phosphate buffer pH 5.5 at 40°C for 48 h. The reaction was stopped by the addition of ice cold 1 M Na_2_CO_3_ (1:1 = v:v) and colorimetric detection of *p*-nitrophenol, the product of *p*NPG hydrolysis, was spectrophotometrically measured at 410 nm (Esen and Blanchard, 2000).

Aerial parts of *C. erythraea* (100 mg) were ground into homogeneous powder using liquid nitrogen and extracted with 1 ml of 99.8% methanol (AppliChem GmbH, Germany) by vortexing for 30 s and subsequent sonication for 10 min using an ultrasonic bath (RK100, Bandelin, Berlin, Germany). After centrifugation at 10,000 *g* for 10 min supernatants were filtered using 0.2 μm syringe filters (Agilent Technologies, Santa Clara, CA, United States). *C. erythrae*a methanol extract (10 μl) was evaporated in a Vacuum Rotor Evaporator (Eppendorf Concentrator 5301, Germany) at room temperature. Subsequently, dried extracts were dissolved in 300 μl 50 mM citrate phosphate buffer (pH = 5.5) containing 20 μg of purified recombinant enzyme (*Ce*BGlu1 or *Ce*BGlu2) and incubated for 48 h at 37°C. Following incubation, 700 μl of methanol was added to stop the reaction, and reaction mixtures were centrifuged for 10 min at 10,000 *g*. Supernatants were filtered through 0.2 μm cellulose filters (Agilent Technologies, United States) and subsequently subjected to UHPLC/DAD/(±)HESI−MS^2^ quantification of SGs. Control samples were prepared by replacing recombinant protein with the elution buffer used for protein purification.

Hydrolytic activity of *Ce*BGlu1 and *Ce*BGlu2 was further tested using standards of epideoxyloganic acid, loganin, secologanin, sweroside, swertiamarin, gentiopicrin, apigetrin, isoquercitrin, and vitexin as substrates. All reference compounds were diluted in dH_2_O (w:v = 1:1) and kept as a stock solutions. Aliquots of 30 μl were dried in vacuum evaporator and diluted in 300 μl 50 mM citrate phosphate buffer pH = 5.5. Reaction mixture containing 30 μl of previously diluted standard compound and 20 μg of purified recombinant enzyme in a final volume of 300 μl 50 mM citrate phosphate buffer pH = 5.5 was incubated for 48 h at 37°C. Subsequently, 700 μl of methanol was added, and samples were centrifuged at 10,000 *g* for 10 min. Control samples contained elution buffer instead of purified recombinant protein, and the final concentration of standards was 3 μg ml^–1^. Supernatants were filtered through 0.2 μm cellulose filters and injected into UHPLC/DAD/(±)HESI−MS^2^ instrument.

### Construction of *Ce*BGlu Expression Plasmids and Agrobacterium-Mediated Transformation

*CeBGlu1* and *CeBGlu2* sequences were PCR-amplified from pTZ57R/T plasmids with the addition of *Pac*I/*Avr*II restriction sites. Amplicons were ligated to transient expression vector pJL-TRBO (Lindbo, 2007), and positive colonies were identified by colony PCR using vector- and gene-specific primers. All PCR reactions were performed with Phusion high-fidelity DNA polymerase (Thermo Fisher Scientific, United States) and a pair of *Ce*BGlu1/*Ce*BGlu2 gene specific primers (Supplementary Table 1). After heat-shock transformation with pJL-TRBO:*Ce*BGlu1 and pJL-TRBO:*Ce*BGlu2 (Figure 2B), Mach1 *E. coli* competent cells were cultured overnight in LB liquid medium containing kanamycin (50 μg ml^–1^) at 37°C in a shaker incubator at 230 rpm (IKA KS 4000 ic control, China). GeneJET Plasmid Miniprep Kit (Thermo Fisher Scientific, United States) was used to purify plasmids from 5 ml of Mach1 *E. coli* cultures according to the manufacturer’s instructions. After plasmid isolation, each *CeBGlu* sequence was confirmed by sequencing.

The recombinant pJL-TRBO:*Ce*BGlu1 and pJL-TRBO:*Ce*BGlu2 plasmids extracted from Mach1 cells were transferred to *A. tumefaciens GV3101* strain by electroporation using “Gene Pulser” (Bio-Rad, United States) and subsequently cultured in LB plates containing kanamycin (50 μg ml^–1^), gentamicine (25 μg ml^–1^) and rifampicin (10 μg ml^–1^). A single colony of recombinant bacteria was inoculated into 5 ml liquid LB media containing antibiotics and incubated overnight at 28°C with shaking at 230 rpm. On the next day, 1 ml of bacterial suspension was sub-cultivated in 10 ml of liquid LB media containing 10 mM MES-KOH (pH 5.5) and 20 μM acetosyringone. Cultures were incubated overnight at 28°C with shaking at 230 rpm. The agrobacterial cells were harvested by centrifugation for 20 min at 3,000 *g*, and the pellet was resuspended in the infiltration medium (10 mM MES, 10 mM MgCl_2_ and 100 μM acetosyringone) to a final OD600 of 1.0. After an incubation at room temperature for 4 h, cultures were introduced into abaxial surface of leaves of five-month-old *C. erythraea* plantlets using a blunt tipped plastic syringe and applying gentle pressure. Additionally, to prevent the silencing of transgene expression in *C. erythraea*, pJL-TRBO:*Ce*BGlu1 and pJL-TRBO:*Ce*BGlu2 were co-infiltrated in a 1:1 ratio with pBIN vector expressing the p19 silencing-suppressor gene from TBSV (pBIN:p19). After agroinfiltration, plants continued to grow for 5 days in the greenhouse. Leaves were harvested from diploid and tetraploid *C. erythraea* plants and immediately frozen at liquid nitrogen. Samples were stored at −80°C until further use.

### Plant Methanol Extract Preparation

Plant material (shoots and roots) of *C. erythraea* was manually ground in liquid nitrogen into fine powder and diluted in 96% methanol (w:v = 10:1). Following vortexing for 1 min, extraction was performed overnight at 4°C. The next day, extraction was continued for 20 min in an ultrasonic bath (RK100, Bandelin, Germany) maintained at room temperature. Samples were centrifuged at 8,000 *g* for 20 min and supernatants were filtered using 15 mm RC filters with 0.22 μm pore size (Agilent Technologies, United States). Samples were stored at 4°C until use. All extractions were performed in biological triplicates.

### UHPLC/DAD/(±)HESI-MS^2^ Quantification of Targeted *β*-D Glucosides

Samples were analyzed using Dionex Ultimate 3000 UHPLC system (Thermo Fisher Scientific, Germany) equipped with a DAD detector and connected to a triple quadrupole mass spectrometer (TSQ Quantum Access MAX, Thermo Fisher Scientific, Switzerland). Samples were chromatographically separated on a Hypersil gold C18 column (50 × 2.1 mm) with 1.9 μm particle size (Thermo Fisher Scientific, United States) thermostated at 40°C. Mobile phase, consisting of water + 0.01% acetic acid (A) and MS grade acetonitrile (B), was eluted at flow rate of 0.4 ml min^–1^ according to Banjanac et al. (2017). Injection volume was set to 10 μl. DAD absorption was acquired at λmax = 260 and 320 nm. A triple quadrupole mass spectrometer with a heated electrospray ionization (HESI) was operated with a following parameters: vaporizer temperature 300°C, spray voltage 4,000 V, sheet gas (N_2_) pressure 27 AU, ion sweep gas (N_2_) pressure 1.0 AU and auxiliary gas (N_2_) pressure at 10 AU, capillary temperature 275°C, skimmer offset 0 V. Argon was used as the collision gas in the collision-induced fragmentation of the selected reaction monitoring (SRM) mode of the instrument, and collision energies (cE) were set as shown in Supplementary Table 3. Calibration curves of targeted compounds showed excellent linearity with correlation coefficients *r* = 0.999, *p* < 0.001. Total concentrations of targeted secoiridoids were obtained by calculating their peak areas, and were expressed as μg per 100 mg of plant fresh weight (μg 100 mg^–1^ FW). Xcalibur software (version 2.2) was used for the instrument control, data acquisition and analysis. All analyses were performed using three biological replicates.

### Statistical Analysis

Statistical significance was determined by using Minitab Statistical Software (Minitab, State College, PA, United States). For statistical analysis of relative gene expression and compound quantification in shoots and roots of diploid and tetraploid plants one-way ANOVA was performed followed by Fisher LSD test (*p* < 0.05). For comparison of hydrolytic activities, as well as for comparing SGs content in leaves after agroinfiltration with *Ce*BGlu1 and *Ce*BGlu2 cloned into pJL-TRBO expression vector (without or with silencing inhibitor p19) Student’s *t*-tests were used for data analysis (*p* < 0.05).

## Results and Discussion

Specific hydrolytic enzymes activate many glycosylated compounds in plants (e.g., glucosinolates, alkaloids, benzoxazinoids, cyanogenic, and (seco)iridoid glucosides), which often defines their dual-defense system against herbivores. Highly active and unstable aglycones released from iridoid and secoiridoid glycosides and monoterpenoid indole alkaloids usually display more prominent biological activities toward herbivores and pathogens, by adversely affecting their enzymatic machinery. Aglycones react with nucleophilic side chains of amino acids to form covalent protein complexes (Bartholomaeus and Ahokas, 1995; Konno et al., 1999; Kim et al., 2000; Guirimand et al., 2010), and act as unspecific enzyme inhibitors (Bartholomaeus and Ahokas, 1995; Ling et al., 2003). The typical defense compounds of Plantaginaceae are iridoid glycosides, which retard growth and/or enhance mortality of non-adapted herbivores. As a part of the dual defense system, *Plantago lanceolata* and *P. major* possess *β*-glucosidases that hydrolyze aucubin, one of the two major iridoid glycosides in these species, and thereby release protein-denaturing aglycones (Pankoke et al., 2013, 2015). Oleaceae species like *Ligustrum obtusifolium* and *Olea europaea* are rich sources of SGs that after tissue disruption are metabolized by endogenous plant *β*-glucosidases (Konno et al., 1999; Mazzuca et al., 2006). Oleuropein *β*-glucosidase (OeGlu) from *O. europea* is reported to have a defensive role in young organs and meristem tissues as well as in mature tissues, where it activates oleuropein into a potent protein cross-linking agent during de-compartmentalization caused by pests (Koudounas et al., 2015). Oleuropein *β*-glucosidase (OeGlu) is highlighted as a molecular target of high biotechnology interest to regulate qualitative and quantitative content of bioactive secoiridoids in olive oils, and thus their organoleptic properties (Koudounas et al., 2021). Likewise, in MIAs-rich *C. roseus* and *R. serpentina* (fam. Apocynaceae), strictosidine is hydrolyzed by *β*-glucosidases (Guirimand et al., 2010). Following the disruption of strictosidine and strictosidine *β*-D-glucosidase (SGD) compartmentalization in *C. roseus*, initiated by cellular breakup after tissue wounding, the highly reactive strictosidine aglycone with prominent feeding-deterrent/toxic properties is released, and further conducted toward the production of cytotoxic MIAs (Guirimand et al., 2010, 2011).

Secoiridoid glucosides of *C. erythraea* are defense compounds constitutively present in tissues that, due to their extremely bitter taste, pre-ingestively deter feeding and thus reduce consumption rates of various non-adapted herbivores. The presumption of the present study was that *C. erythraea* might also possess *β*-glucosidases displaying high specificity toward SGs. By activating aglycones of SEC, SW, SWM, and GP, these hydrolytic enzymes could be involved in initial steps of their catabolism. Furthermore, resulting aglycones might offer multi-level protective features to plants, and, as previously shown, shape their antioxidant properties (Božunović et al., 2018).

### Isolation of Full Length of *Ce*BGlu, Comparison With Homologs, and Phylogenetic Analysis

**FIGURE 3:**
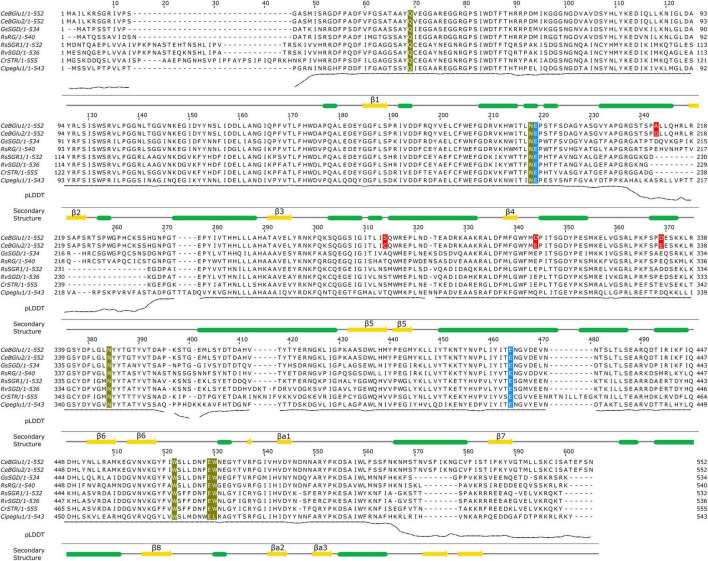
Multiple sequence alignment of *Ce*BGlu1 and *Ce*BGlu2 with *β*-glucosidases from species belonging to the Gentianales order. The two Glu catalytic residues are colored blue, active site residues involved in sugar specificity are colored olive, and residues differing between *Ce*BGlu1 and *Ce*BGlu2 are indicated in red. The secondary structure diagram shown below the alignment is based on the *Ce*BGlu1 AlphaFold model. The pLDDT score (predicted lDDT-Cα) shown below the alignment is a per-residue estimate of AlphaFold confidence on a scale from 0 to 100. Lower values are often associated with disordered regions. It is shown for *Ce*BGlu1 AlphaFold model. RsRG sequence corresponds to the PDB:3U5Y glucosidase from *Rauvolfia serpentina.*

By exploring transcriptomic resources of *C. erythraea* leaves, we identified a candidate for *β*-D- glucosidase (*Ce*BGlu) displaying high homology with strictosidine *β*-D-glucosidase previously characterized from *C. roseus* (Geerlings et al., 2000). Following PCR, cloning of amplified product into *pTZ57R/T* vector and sequencing, two highly similar *Ce*BGlu candidates (*Ce*BGlu1 and *Ce*BGlu2) were revealed. Full lengths of isolated *CeBGlu1* and *CeBGlu2* comprised open reading frames of 1,659 bp encoding polypeptides of 552 amino acids with calculated molecular masses of 62.06 kDa. The two *Ce*BGlu gene candidates shared a high level of similarity (99.3%) and differed at 12 nucleotide sites, i.e., 4 amino acids (Figure 3 and Supplementary Table 4). Based on primary structure both enzymes were predicted to be localized in the lysosome/vacuole.

Amino acid sequences of *Ce*BGlu1 and *Ce*BGlu2 were aligned with previously investigated *β*-D-glucosidase amino acid sequences of plant species belonging to the Gentianales order (Figure 3): strictosidine glucosidase from *Gelsemium sempervirens* (*Gs*SGD) (Franke et al., 2019), raucaffricine-*O*-*β*-D-glucosidase (*Rs*RG) (Warzecha et al., 2000) and strictosidine *β*-D-glucosidase (*Rs*SGR1) from *Rauvolfia serpentina* (Xia et al., 2012), strictosidine *β*-D-glucosidase from *R. verticillata* (*Rv*SGD) (Chen et al., 2008), strictosidine *β*-D-glucosidase from *C. roseus* (*Cr*STR) (Geerlings et al., 2000), and beta-glucosidase from *Carapichea ipecacuanha* (*Ci*Ipeglu1) (Nomura et al., 2008). The highest amino acid sequence identity for *Ce*BGlu1 and *Ce*BGlu2 was observed with *GsSGD* (69.2 and 69.8%, respectively) (Supplementary Table 4).

**FIGURE 4:**
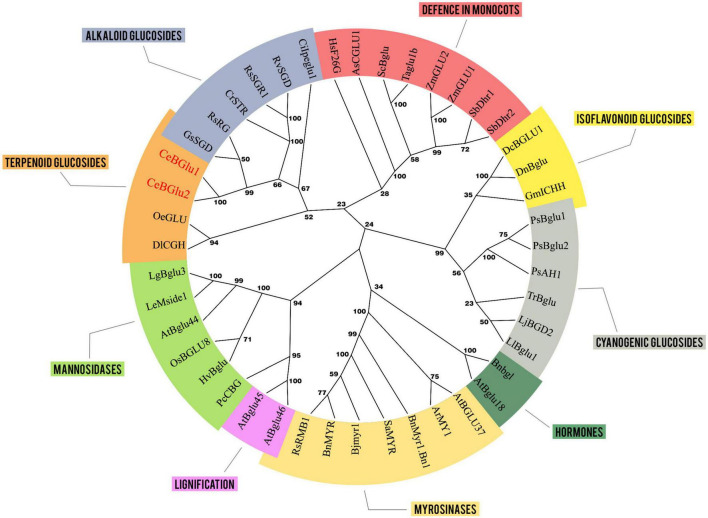
The neighbor-joining tree generated using MEGA X (Version 10.2.6) with a bootstrap of 1,000 replicates, illustrating the phylogenetic relationship of the *Ce*BGlu1 and *Ce*BGlu2 candidates (red letters) relatively to homologous genes from various plant species (refer to Supplementary Table 2 for a list of plant protein sequences used for phylogenetic analysis and their corresponding accession numbers). The optimal tree is shown. The percentage of replicate trees in which the associated taxa clustered together in the bootstrap test (1,000 replicates) are shown next to the branches. Groups of enzymes with experimentally defined functions are labeled with different colors.

Phylogenetic tree was constructed incorporating *β*-D-glucosidase amino acid sequences of diverse plant species to narrow the prediction of the function of both *Ce*BGlu candidates (Figure 4). The neighbor-joining tree grouped the enzymes into several clusters based on their role in plants: defense response, lignification, hormone deglycosylation, *β*-mannosidase or myrosinase activities. Defense-related enzymes included a separate cluster that belonged to monocots, and four clusters belonging to dicots, having different types of substrates: cyanogenic glucosides, isoflavonoid conjugates, alkaloid glucosides, and terpenoid glucosides. Interestingly, even though their substrates belong to the group of terpenoids, the two *C. erythraea* candidates (*Ce*BGlu1 and *Ce*BGlu2) showed the highest homology with *β*-D-glucosidases of the order Gentianales that prefer alkaloid glucosides as substrates (*Gs*SGD, *Rs*RG, *Rs*SGR1, *Rv*SGD, *Cr*STR, and *Ci*Ipeglu1) (Figure 4).

### Comparative Analysis of Organ-Specific Secoiridoid Glucosides Content and *Ce*BGlu Expression and Activity

**FIGURE 5:**
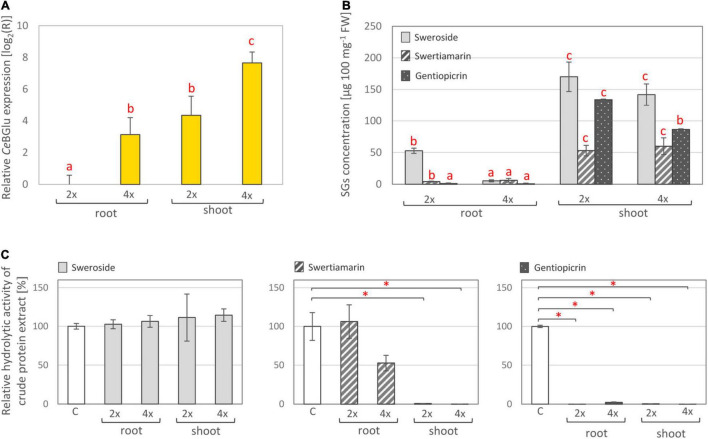
**(A)** Relative expression of *CeBGlu* in shoots and roots of *in vitro* grown *C. erythraea* diploid (2×) and tetraploid (4×) plants. Values are normalized to the *CeBGlu* expression level in roots of diploid plants. Letters above the bars denote significant differences according to Fisher LSD test at *p* < 0.05. **(B)** Content of major secoiridoids in shoots and roots of diploid and tetraploid *C. erythraea* plants. Values represent means ± SE (*n* = 3). Letters above the bars denote significant differences according to Fisher LSD *post hoc* test at *p* < 0.05, for each compound independently. **(C)** Hydrolytic activity of total proteins isolated from shoots and roots of diploid and tetraploid *C. erythraea* genotypes, using sweroside, swertiamarin and gentiopicrin as substrates. The bars indicate the amount of substrates (%) not enzymatically hydrolyzed in an *in vitro* enzymatic assay. Control group (white bars), where no protein extract was added, was set to 100%, and all the values are presented relatively, in respect to control. Data are mean ± SD (*n* = 3). Red asterisks denote significantly different adjusted *p*-values, *p < 0.001.

Plant glucosidase genes are developmentally regulated (Morant et al., 2008a), and exhibit different spatial expression patterns depending on their physiological functions. In this sense, we analyzed the organ-specific distribution of transcript levels and activities of the two *C. erythraea *β**-glucosidase candidates, in parallel with the content of major SGs, in both diploid and tetraploid genotypes. The presence of *Ce*BGlu1 and *Ce*BGlu2 transcripts in *C. erythraea* was validated by amplifying their specific fragments using qRT-PCR. Due to the high sequence similarity between the two gene candidates, a combination of primers common to both transcripts were employed (Figure 5A). *CeBGlu* was amplified at low level when cDNA from roots was used as a template in qRT-PCR analysis. Expression analysis revealed that the transcription of *CeBGlu* is regulated in an organ-specific manner (Figure 5A). The *CeBGlu* expression pattern revealed significantly higher transcript levels in shoots compared to roots of *C. erythraea*, in both diploid and tetraploid genotypes. However, tetraploid plants displayed significantly higher *CeBGlu* transcript levels in shoots and roots compared to diploids.

In parallel, content of major secoiridoids (SW, SWM, and GP) was significantly higher in shoots than in roots of both diploid and tetraploid *C. erythraea* genotypes (Figure 5B). It is well documented that aerial parts are the major site of SGs biosynthesis and accumulation in common centaury (Šiler et al., 2012, 2014; Matekalo et al., 2018; Božunović et al., 2019; Filipović et al., 2019). Similarly, as in our previous study (Božunović et al., 2019), the dominant SG in leaves of diploid and tetraploid plants was SW, followed by GP and SWM. Diploid plants displayed slightly higher GP content in shoots than tetraploid ones, which is in accordance with our previous study (Filipović et al., 2019), and higher content of SW in roots compared to tetraploids.

To test the total hydrolytic activity of *C. erythraea* organs, pure SW, SWM and GP were subjected to an *in vitro* enzymatic assay using crude protein extract of shoots or roots, and the decrease in SG content was analyzed using UHPLC/DAD/(±)HESI−MS^2^ analysis. SWM and GP were efficiently hydrolyzed, while no significant decrease in SW content was recorded (Figure 5C). Shoots of both diploid and tetraploid genotypes displayed more intensive hydrolysis of SWM than corresponding roots. Both shoots and roots were efficient in hydrolyzing GP. These results generally indicate higher SG-related *β*-D-glucosidase activity in shoots than in roots, which corresponds to higher expression level of the two candidates identified within the present study.

Lower content of SGs in shoots and roots of tetraploid individuals when compared to diploids might be, at least partially, ascribed to higher expression level of SGs-related *β*-D-glucosidases and higher activity of these enzymes in tetraploids. Tetraploids most likely display more intense SGs catabolism which is reflected through lower SGs content. As tetraploid plants used in the present study are not a direct offspring of the diploid ones, the observed divergence between them is most likely also influenced by differences in their genomes.

### Cloning and Heterologous Expression of *Ce*BGlu in *E. coli*

Isolated BGlu candidates were sub-cloned into the pRSETA vector; pRSETA:*CeBGlu1* and pRSETA:*CeBGlu2* constructs were transformed into *E. coli* BL21 (DE3) competent cells. To achieve simple and efficient purification of the enzyme, *CeBGlu1* and *CeBGlu2* were expressed in fusion with the His-tag.

**FIGURE 6:**
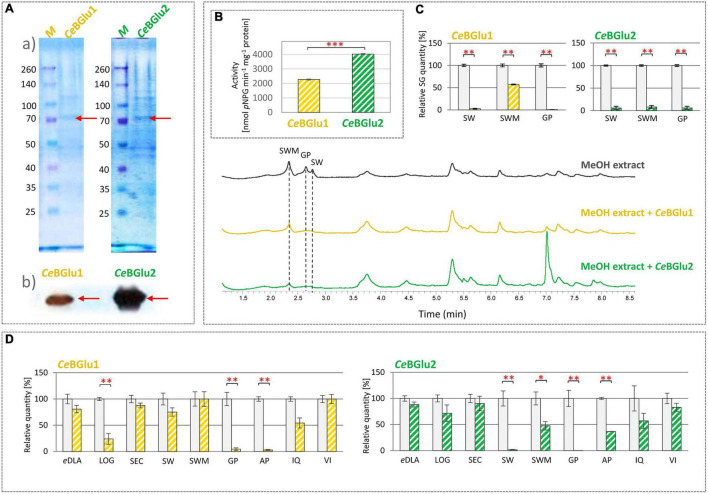
**(A)** Characterization of recombinant *Ce*BGlu1 and *Ce*BGlu2 produced in *E. coli* BL21: **(a)** SDS-PAGE electropherogram; M- pre-stained SDS-PAGE size marker; purified recombinant *Ce*BGlu1 and *Ce*BGlu2 eluted after His-tag affinity purification, **(b)** Immunodetection of recombinant *Ce*BGlu1 and *Ce*BGlu2 using anti-His antibodies. **(B)** Hydrolytic activity of recombinant *Ce*BGlu1 and *Ce*BGlu2 against synthetic *p*NPG substrate. **(C)** Hydrolytic activity of *Ce*BGlu1 and *Ce*BGlu2 toward secoiridoids present in methanol extracts of *C. erythraea*. Presented are representative UHPLC/DAD chromatograms of *C. erythraea* methanol extracts at λ = 260 nm, before (black line) and after the hydrolysis with *Ce*BGlu1 (orange line) and *Ce*BGlu2 (green line). **(D)** Hydrolytic activity of recombinant *Ce*BGlu1 and *Ce*BGlu2 against 1,5,9-epideoxiloganic acid (*e*DLA), loganin (LOG), secologanin (SEC), sweroside (SW), swertiamarin (SWM), gentiopicrin (GP), apigetrin (AP), isoquercitrin (IQ), vitexin (VI). The bars indicate the quantity of substrates (%) not enzymatically hydrolyzed in an *in vitro* enzymatic assay. Control group, where no protein extract was added, was set to 100%, and all the values are presented relatively, in respect to control. Data are mean ± SD (*n* = 3). Red asterisks denote significantly different values according to the *t*-test, *p*-values, *p < 0.05, **p < 0.01, ***p < 0.001.

Following expression of recombinant proteins in *E. coli*, they were isolated using His-tag affinity purification and subsequently resolved in SDS-PAGE (Figure 6Aa and Supplementary Figure 1). SDS-PAGE analysis of the purified recombinant proteins detected bands with a slightly higher *Ce*BGlu1 and *Ce*BGlu2 molecular masses of ∼70 kDa than expected (62.06 kDa) (could be, at least partially, ascribed to a His-tail of recombinant proteins). Purified preparations of recombinant *Ce*BGlu1 and *Ce*BGlu2 were also analyzed by western blot using the anti-6×His antibody (Figure 6Ab). In both cases, a protein band with the same molecular mass as deduced from the SDS-gel was observed.

### *In vitro* Functional Characterization of *Ce*BGlu1 and *Ce*BGlu2

Substrates such as 4-nitrophenyl-*β*-D-galactopyranoside, 4-nitrophenyl-*β*-D-thioglucoside, and 4-nitrophenyl-*β*-D-glucopyranoside (*p*NPG), are most commonly used artificial substrates in enzymatic assays for confirmation of *β*-glucosidase enzymes function (Czjzek et al., 2000; Geerlings et al., 2000; Warzecha et al., 2000; Morant et al., 2008b; Shaik et al., 2013; Tiwari et al., 2016). In order to examine functionality of *Ce*BGlu1 and *Ce*BGlu2, their hydrolytic activity against commercial substrate 4-nitrophenyl-*β*-D-glucopyranoside (*p*NPG) was analyzed. Both enzymes efficiently hydrolyze synthetic glucoside *p*NPG, which confirms their glucosidase activity (Figure 6B). *Ce*BGlu2 exhibits a higher affinity for the commercial *p*NPG substrate (4.029 μmol pNPG min^–1^ mg^–1^ protein) compared to *Ce*BGlu1 (2.267 μmol *p*NPG min^–1^ mg^–1^ protein). Similarly, enzymes isolated from *Lamium galeobdolon* (*Lg*Glu2 and *Lg*Glu4) hydrolyze *p*NPG, but the efficiency of the two tested enzymes differs (Hannemann et al., 2018). Purified *β*-glucosidases of *Lotus japonicus* (*Lj*BGD2 and *Lj*BGD4) hydrolyze *p*NPG, but show higher affinity for plant-synthesized substrates, such as prunasin, lotaustralin, and rhodiocyanoside (Morant et al., 2008b). However, plant *β*-glucosidases are not always effective in reaction with synthetic substrates. Thus, *β*-glucosidase of *O. europaea* (*Oe*Glu) does not show hydrolytic activity against tested *p*NPG substrate, and its function was assigned based on a reaction in which *Oe*Glu efficiently hydrolyzes a specific substrate available *in planta* – secoiridoid oleuropein (Koudounas et al., 2015). Also, *β*-glucosidases of *Arabidopsis thaliana* (AtBGlu21, AtBGlu22, and AtBGlu23) hydrolyze the tropan alkaloid scopolin, but do not exhibit hydrolytic activity against a synthetic substrate (Ahn et al., 2010).

*Ce*BGlu1 and *Ce*BGlu2 hydrolytic activity was subsequently tested in enzymatic reactions with SG-rich methanol extract of *C. erythraea* as a substrate, and changes in the content of major SGs (SW, SWM, and GP) were recorded (Figure 6C). *Ce*BGlu2 was more efficient than *Ce*BGlu1 in hydrolyzing SWM, which was the most abundant SG in *C. erythraea* methanol extract used in experiments. *Ce*BGlu1 and *Ce*BGlu2 were equally efficient against SW and GP. As the amounts of SW (2.0 μg ml^–1^ extract), SWM (3.5 μg ml^–1^ extract) and GP (2.1 μg ml^–1^ extract) in *C. erythraea* methanol extract were not equal, it was not possible to make conclusions about the preferable substrates of the two *Ce*BGlu.

To test the substrate specificity, *β*-D-glucosidase activity of recombinant *Ce*BGlu1 and *Ce*BGlu2 enzymes was determined against several plant-derived glucosides of the *β*-D-type (Figure 6D). Hydrolytic activity of *Ce*BGlu1 and *Ce*BGlu2 was tested against iridoid glucosides (1,5,9-epideoxyloganic acid- 1,5,9-eDLA and loganin- LOG), secoiridoid glucosides (SEC, SW, SWM and GP), and flavonoid glucosides (apigetrin- AP, isoquercitrin- IQ, and vitexin- VI). Pure 1,5,9-eDLA was isolated from methanol extracts of *Nepeta rtanjensis* as described by Aničić et al. (2021), while AP and VI, as well as standards of iridoids and secoiridoids, were commercially purchased. As standards for aglycones of secoiridoids were not available, the *β*-D-glucosidase activity of recombinant proteins was evaluated by UHPLC/DAD/(±)HESI−MS^2^ quantification of targeted glucosides (Supplementary Figure 2). Their content in reaction mixtures were compared to those of the negative controls where recombinant proteins were excluded. Although two recombinant proteins share 99.3% similarity of amino acid sequences, *Ce*BGlu1 and *Ce*BGlu2 display slightly differential hydrolytic activity and specificity against tested substrates (Figure 6D). The highest activity of *Ce*BGlu1 was recorded when using GP and AP as substrates, which were hydrolyzed by 95.4 and 97.1%, respectively. This enzyme was also efficient in hydrolyzing LOG (76.0%). The highest hydrolytic activity of *Ce*BGlu2 was recorded for GP (99.7%) and SW (98.3%), followed by AP (63.5%) and SWM (52.2%). Generally, both *Ce*BGlu1 and *Ce*BGlu2 display higher affinity for GP, than for SW and SWM. Although significant activity of *Ce*BGlu1 and *Ce*BGlu2 was recorded when using AP (apigenin 7-O-glucoside) as a substrate, one should bear in mind that, up to the best of our knowledge, this compound was not previously detected in *C. erythraea* methanol extracts (Šiler and Mišić, 2016; Banjanac et al., 2017; Božunović et al., 2018). Slight hydrolytic activity of *Ce*BGlu1 and *Ce*BGlu2 with 1,5,9-*e*DLA, SEC and IQ was recorded, but it was not statistically significant (Figure 6D). The two enzymes displayed no hydrolytic activity against VI, an apigenin 8-C-glycoside, which was also not previously detected in *C. erythraea*. *β*-D-glucosidases display substantial substrate promiscuity (Hannemann et al., 2018) so the hydrolysis of several substrates is not uncommon. Many beta-glucosidases have transglucosidase activities in addition to their hydrolase activity (Opassiri et al., 2004). However, the number of glucoconjugates in plants is likely larger than the number of beta-glucosidases and the enzymes tend to have overlapping specificities, which complicates determination of their exact functions. These cases of multiple functions have been categorized into “multitasking,” where the enzyme carries out multiple functions at the same time, or “moonlighting,” where the enzyme has two different functions in divided situations, according to Ketudat Cairns et al. (2015).

### Transient Overexpression of *Ce*BGlu1 and *Ce*BGlu2 in Leaves of Diploid and Tetraploid *Centaurium erythraea*

Transient expression of recombinant proteins in plants using tobacco mosaic virus (TMV) based viral vectors have been well documented (Chiong et al., 2021). TMV-based overexpression pJL-TRBO vector can be used for the insertion of a target gene by agroinfiltration into plants. *Agrobacterium*-mediated delivery of genes, results in highly efficient transient expression of the foreign protein, producing up to 100-fold more recombinant protein compared to a non-viral system (Lindbo, 2007).

**FIGURE 7:**
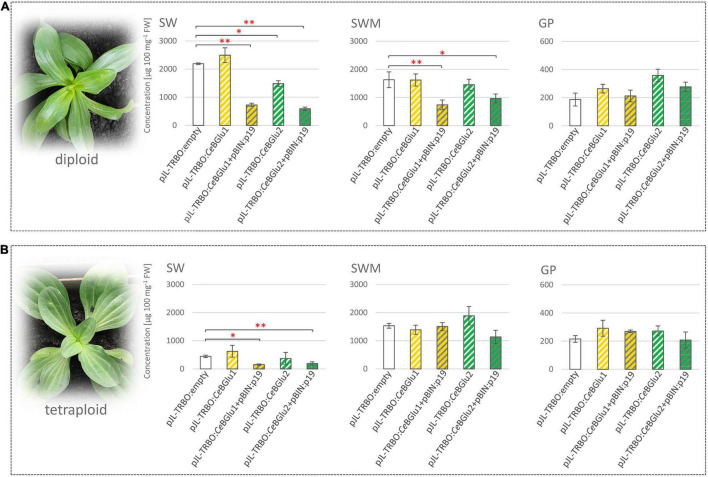
Agroinfiltration of leaves of diploid **(A)** and tetraploid **(B)** *C. erythraea* plants. Changes in the content of secoiridoid glucosides (SGs) swertiamarin (SWM), sweroside (SW) and gentiopicrin (GP) 5 days following agroinfiltration were recorded: *Ce*BGlu1 and *Ce*BGlu2 cloned into pJL-TRBO expression vector, were co-expressed either without or with silencing inhibitor p19 (pBIN:p19 construct). Results are expressed as means ± SE. Red asterisks denote significantly different values according to the *t*-test, *p*-values, *p < 0.05, **p < 0.01.

To verify the functional identity of the *Ce*BGlu gene, *Agrobacterium*-mediated transient expression in centaury leaves was carried out. To that end, the *Ce*BGlu coding region was subcloned into the pJL-TRBO vector. The resultant plasmids designated pJL-TRBO:*Ce*BGlu1 and pJL-TRBO:*Ce*BGlu2 were used to transform *A. tumefaciens* GV3101 for the transient *in planta* expression. The two *Ce*BGLU candidates were overexpressed in leaves of five-month-old diploid and tetraploid *C. erythraea* plants, alone or in combination with p19 (Figure 7). Phytochemical profiling of SGs in leaves of diploid and tetraploid *C. erythraea* plants, harvested 5 days after agroinfiltration, was performed. Overexpression of *Ce*BGlu1 and *Ce*BGlu2 induced changes of SGs content in leaves of diploid and tetraploid *C. erythraea* plants when co-expressed with p19 gene silencing-suppressor (Figure 7). In general, phytochemical changes were more pronounced in leaves of diploid plants. Significant decrease in SWM content was recorded in diploid plants jointly over-expressing *Ce*BGlu1 and p19, or *Ce*BGlu2 and p19. The content of SW in diploid plants was significantly decreased following overexpression of *Ce*BGlu1 and *Ce*BGlu2 in combination with p19, but also when *Ce*BGlu2 was over-expressed alone. In tetraploid plants, SW content was decreased following agroinfiltration with *Ce*BGlu1 and *Ce*BGlu2 in combination with p19. No statistically significant alterations in GP content in response to agroinfiltration with *Ce*BGlu1 and *Ce*BGlu2 was observed, in both diploid and tetraploid plants. Viral-encoded suppressor of gene silencing, the p19 protein of tomato bushy stunt virus (TBSV), which is believed to prevent the onset of post-transcriptional gene silencing in the infiltrated tissues (Voinnet et al., 2003; Shah et al., 2013), obviously allowed high level of *Ce*BGlu1 and *Ce*BGlu2 transient expression, which resulted in pronounced phytochemical changes in *C. erythraea* leaves. Previous studies have suggested that transgene expression or RNA silencing in plants can be affected by ploidy, and is significantly less efficient in tetraploids than in diploids (Finn et al., 2011). In other words, transgenes are more prone to transcriptional inactivation in polyploids than in diploids (Mittelsten Scheid et al., 1996, 2003), which often poses a major limitation to polyploids improvement via biotechnology (Gao et al., 2013). Thus, it is not surprising that the use of p19 more efficiently increases the transient expression of recombinant *Ce*BGlu1 and *Ce*BGlu2 proteins in leaves of *C. erythraea* diploids than in tetraploids, which is accompanied with the more pronounced decrease in SGs content (Figure 7). Taken together, overexpression of the two *CeBGlu* isogenes decreased the content of SGs in *C. erythraea* leaves, which additionally confirmed the *β*-glucosidase function of these two genes. The entire centaury genome sequence is still unknown, yet it probably contains many other *β*-glucosidases of diverse as well as similar functions and substrate specificities, which could also contribute to shaping the secoiridoid profiles of these remarkable plants.

### Comparative 3D Modeling of *Ce*BGlu1 and *Ce*BGlu2

**FIGURE 8:**
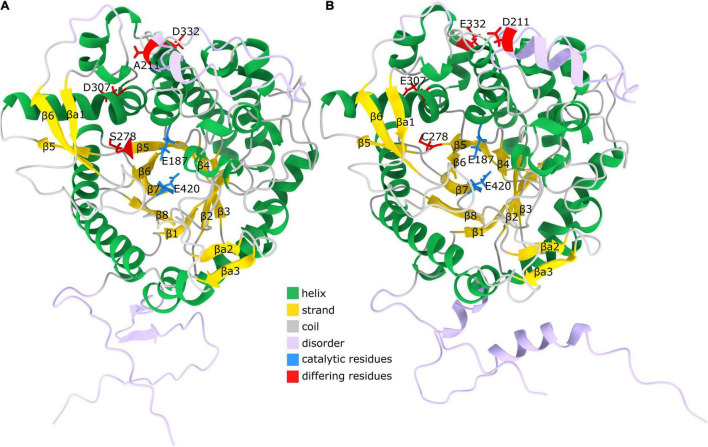
Predicted structure of *Ce*BGlu. **(A)** AlphaFold model of *Ce*BGlu1. Helices are shown in green, strands in gold and coils in gray. Regions with pLDDT < 80 are indicated with lavender color. The two active site residues Glu187 and Glu420 are indicated with blue color, while residues differing between *Ce*BGlu1 and *Ce*BGlu2 are indicated in red **(B)** AlphaFold model of *Ce*BGlu2. Coloring is as under **(B)**.

To gain insights into sequence-structure relations of the two centaury *β*-glucosidases their 3D structure was inferred using AlphaFold (models provided as Supplementary Material 1). The obtained models had a low percentage of Ramachandran outliers and a low clash score (Supplementary Table 5). The percentage of Ramachandran favored Cα angles was between 92 and 93% for the two models. The majority of residues not within the favored parts of the Ramachandran diagram had a low pLDDT value (a per-residue estimate of AlphaFold confidence on a scale from 0 to 100) suggesting they are in disordered regions. The two predicted models corresponding to *Ce*BGlu1 to *Ce*BGlu2 are highly structurally similar, especially if the likely disordered regions are not taken into account – the RMSD of the superimposed pruned 475 Cα atoms in the two structures is 0.216 Å. Both obtained models showed a high degree of structural similarity to the experimental structure of the raucaffricine *β*-D-glucosidase from *R. serpentina* (PDB: 3U5Y, Supplementary Table 5; Xia et al., 2012). The models have a (*β*/α) 8-barrel (Figures 8A,B) fold characteristic for the CAZy GH1, consisting of eight parallel *β*-strands forming a barrel-like structure. These strands are connected via intricate helical regions and loops (Figure 8). Strands *β*5 and *β*6 are longer compared to other, and protrude the barrel (Figures 8A,B). Based on AlphaFold secondary structure annotation these are split into two strand regions each. Together with an antiparallel strand *β*a1 (Figure 8) which is not a part of the barrel, *β*5 and *β*6 form a sheet. After the (*β*/α) 8-barrel structure is a sheet formed from two antiparallel strands *β*a2 and *β*a3. The regions with low pLDDT are on the termini, between *β*4 and *β*5, as well as between *β*6 and *β*a1, matching closely the regions with missing density in the experimental structure of raucaffricine *β*-D-glucosidase from *R. serpentina* which strengthens the conclusion these regions are disordered.

**FIGURE 9:**
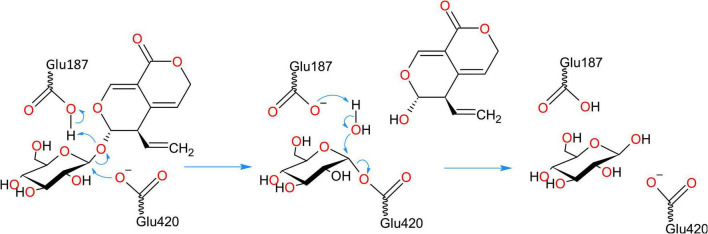
Retaining double displacement reaction mechanism of GH1 *β*-glucosidase enzymes. Glu187 is the catalytic proton donor which activates the leaving group while Glu420 residue is the nucleophile. The reaction proceeds via a covalent glycosyl enzyme intermediate with an inverted bond which is hydrolyzed by nucleophilic attack of a water molecule, activated by the proton donor residue, resulting in second bond inversion and in overall anomeric retention.

The enzymes from GH1 family have a retaining double displacement mechanism in which one Glu residue acts as a proton donor for the leaving group, while another Glu residue acts as a nucleophile (Ketudat Cairns and Esen, 2010). The nucleophilic attack forms a covalent glycosyl enzyme intermediate with an inverted bond (Figure 9). This covalent intermediate is hydrolyzed by nucleophilic attack of a water molecule, activated by the proton donor residue, resulting in second bond inversion and in overall anomeric retention. The catalytic proton donor and nucleophile in the two centaury *β*-glucosidases correspond to Glu187 and Glu420, respectively (Figure 9). Glu187 is located in the coil just after *β*4, while Glu420 is situated at the end of *β*7, while their Cα atoms are positioned at a distance of 10.3 Å in the models. The pKa values of respective carboxylic groups were estimated to be ∼8 for Glu187 and ∼5.5 for Glu420 which is in concordance to their proposed role of proton donor and nucleophile, respectively.

**FIGURE 10:**
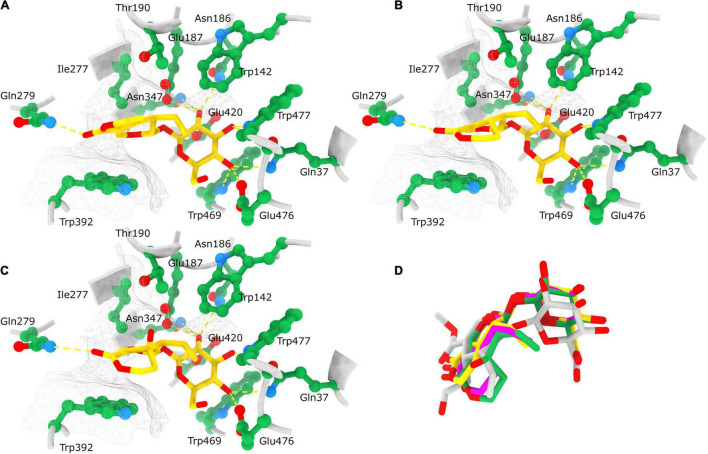
Position of docked ligands in the *Ce*BGlu1 active site. Interactions of docked ligands: gentiopicrin **(A)**, sweroside **(B)** and swertiamarin **(C)** with the *Ce*BGlu1 active site residues. Ligands are colored yellow with oxygen shown in red; active site residues are colored green with oxygen shown in red and nitrogen in blue. H-bonds are indicated with yellow dashed lines while the hydrophobic surface encompassing the aglycone is indicated with a silver mesh. **(D)** Superposition of ligands docked to the *Ce*BGlu1 active: gentiopicrin (yellow), sweroside (pink) and swertiamarin (green) with secologanin (gray) from the experimental structure of glucosidase from *Rauvolfia serpentina* (PDB: 3U5Y). Superposition of *Ce*BGlu1 with 3U5Y was performed using matchmaker command in ChimeraX using default options.

The two centaury *β*-glucosidases differ in four amino acids which include, from *Ce*BGlu1 to *Ce*BGlu2: A211D, S278C, D307E and D332E; the first two being radical replacements, while the latter two are conservative replacements. Position 211 is located in the disordered region between *β*4 and *β*5; 278 is located in *β*5, proximate to, but with a side chain facing away from the active site, while 307 and 332 are located in the helical regions connecting *β*5 to *β*6 (Figure 8). Based on the type, position and orientation of the differing amino acids at positions 211, 307, and 332 no impact of the mentioned amino acids replacements on the overall enzyme performance can be expected. It should be noted that these amino acids are not at the dimer interface based on comparison with 3U5Y (result not shown), so not even a long-distance interaction, due to differential multimer binding, is to be expected. However, one cannot rule out the possible indirect roles of these amino acids on the overall enzyme performance. On the other hand, the position 278 is close to the substrate binding site, and the amino acids around it – Ile277 and Gln279 are directly involved in interactions with the aglycone (Figure 10). Thus, even though the predicted structures of *Ce*BGlu1 to *Ce*BGlu2 are highly similar, including the active site, and the orientation of Ile277 and Gln279 side chains, S278C could have an impact on substrate recognition and overall catalytic performance. It should be noted there are no nearby Cys with which potential disulfide bonds can be formed by Cys287. Thus, the observed differences in the substrate preferences of the two *Ce*BGlu enzymes (Figure 6), might, at least partially, be ascribed to the differences in their amino acid sequences, especially at position 287.

To explore and visualize the binding of substrates to the active site ligand docking was analyzed only for *Ce*BGlu1, because of the very high overall similarity of the predicted models for the two *β*-glucosidases. To paraphrase quantitatively, the RMSD between the 236 atom pairs belonging to residues Gln37, Trp142, Asn186, Glu187, Thr190, Ile277, Gln279, Asn347, Trp392, Glu420, Trp469, Glu476, and Trp477 which form the active sites including the catalytic residues between the two predicted structures is 0.625 Å. Most open-source software for ligand docking is able to perform some sort of conformer search during docking, usually limited to rotatable bonds. The new AutoDock-Vina version implements a macrocycle conformer sampling method (Eberhardt et al., 2021), however, an analogs method appropriate for small aliphatic rings is lacking. Due to this, sets of conformers were pre generated by the ETKDG3 method with small ring torsion angle preferences (Wang et al., 2020) for GP, SW and SWM and used for docking. Prior to docking, while comparing the centaury *β*-glucosidase models with similar experimental structures from the PDB it was noticed *Ce*BGlu1 and *Ce*BGlu2 Glu476 side chain clashes with the glucose in the active center of superimposed experimental structures. Therefore, a flexible docking procedure was employed where Glu476 was allowed to change conformations, while all other enzyme residues were rigid. For GP the highest scoring docked pose (Table 1 and Figure 10A) seems to be very close to what could be expected *in vivo*, at least based on comparison to the pose of the secologanin ligand in the experimental structure of raucaffricine *β*-D-glucosidase from *R. serpentina* (Figure 10D, 3U5Y, Xia et al., 2012). The specificity of the active site for *β*-glucosides is achieved by numerous H-bonds with the sugar OH groups: Asn186, Glu187 and Asn347 form H-bonds with O2, Gln37 and Trp477 with O3, Gln37, Trp469 and Glu476 with O4 on *β*-glucose (Figure 10A). The aglycone binding pocket is tight and hydrophobic, enclosed from one side by Trp392 which is parallel to the aglycone, and from the others by Trp142, Thr190 and Ile277. The only polar interaction with the ligand is via an H-bond between Gln279 and the keto oxygen. The relatively tight space in the aglycone binding site allows mostly planar aglycones, while Gln279 position favors aglycons with H-acceptors or H-donors in the appropriate position. The positions of the highest scoring poses for SW and SWM were not suitable for hydrolysis, because the aglycone occupied the position of glucose. However, the 2nd best pose for SWM and 3rd best pose for SW (Table 1 and Figures 10B,C), which scored only slightly worse, were very similar both to the previously mentioned docked GP, as well as to secologanin ligand in 3U5Y (Figure 10D). The suboptimal highest scoring poses for SWM and SW might be the result of limitations of the used docking procedure, and imperfections in the scoring function.

**TABLE 1:** Ligand docking summary into *Ce*BGlu1 AlphaFold model.

Ligand	Chosen pose affinity (kcal/mol)	Best pose affinity (kcal/mol)	Rank of chosen pose
Gentiopicrin	−9.561	−9.561	1
Swertiamarin	−9.105	−9.169	2
Sweroside	−9.359	−9.644	3

## Conclusion

The present study highlights the direct link between *β*-D-glucosidases characterized within the present study (*Ce*BGlu1 and *Ce*BGlu2) and the content of SGs in analyzed *C. erythraea* tissues. Thus, it provides the first evidence of SGs-related *β*-glucosidases in centaury plants, and advances our understanding of SGs deglycosylation, as a part of their catabolism. These enzymes could serve as a molecular target of high biotechnological interest, in order to produce centaury plants with an optimal composition of secoiridoids, which are essential defense compounds in plants with beneficial effects in human and animal health. One of the major challenges ahead is to gain better understanding of how plants regulate the function of *Ce*BGlu1 and *Ce*BGlu2 in distinct tissues and developmental stages, and in response to biotic and abiotic stress stimuli, which is the course of our further work. Furthermore, it is imperative to assess their biological function and confirm their localization at the cellular and organelle level and define conditions under which these enzymes come into contact with their physiological substrates. Alternatively, gene silencing of the *Ce*BGlu1 and *Ce*BGlu2 combined with metabolic profiling of silenced plants might provide more information on the role and function of these genes, and could also result in elevated amounts of SGs in tissues. In parallel, unrevealing the remaining unknown steps of SGs biosynthetic pathway in *C. erythraea* is of essential importance for establishing the biotechnology-based production of these valuable bioactive compounds. Accumulating knowledge will, in the future, enable manipulation of SGs biosynthesis and catabolism through multi-target metabolic engineering, which will further enable large-scale production of desired secoiridoids in *C. erythraea*.

## Data Availability Statement

The data presented in this study are deposited in the National Center for Biotechnology Information (nih.gov) repository (NCBI; https://www.ncbi.nlm.nih.gov/) under accession numbers ON060690 and ON060691.

## Author Contributions

JB, DMa, SŽ, MM, MS, and DMi conceived and designed the experiments. JB, MM, NA, MS, DMa, SŽ, BF, and TB performed the experiments. DMi performed the phytochemical characterization of samples. MD was responsible for the 3D modeling and ligand docking analysis. JB, MM, DMa, and DMi organized and wrote the manuscript with editing from all the authors. All authors contributed to the article and approved the submitted version.

## Conflict of Interest

The authors declare that the research was conducted in the absence of any commercial or financial relationships that could be construed as a potential conflict of interest.

## Publisher’s Note

All claims expressed in this article are solely those of the authors and do not necessarily represent those of their affiliated organizations, or those of the publisher, the editors and the reviewers. Any product that may be evaluated in this article, or claim that may be made by its manufacturer, is not guaranteed or endorsed by the publisher.

## Footnotes

1
http://www.bioinformatics.nl/cgi-bin/primer3plus/primer3plus.cgi

2
https://www.rcsb.org/alignment
