# Supplementary material for: Functional Characterization of Genes Coding for Novel β-D-Glucosidases Involved in the Initial Step of Secoiridoid Glucosides Catabolism in Centaurium erythraea Rafn
Source: Front Plant Sci. 2022 Jun 23;13:914138. doi: 10.3389/fpls.2022.914138 (PMC9260424; doi:10.3389/fpls.2022.914138)
Supplement: Supplementary file 2 [file Table_1.DOCX]

**Supplementary Table 1.** Sequences of primers used within the present study.

|  | Primer | Sequence (5’ to 3’) |
| --- | --- | --- |
| *1* | *Ce*BGlu_full lenght_F* | GCTTAATCGGCTGAAATGGCA |
| *2* | *Ce*BGlu_full lenght_R | TACATTAATTTGAAAATTCTGTTGCAG |
| *3* | *Ce*BGlu_qPCR_F | TTTCCGCCAGTACGTAGAGC |
| *4* | *Ce*BGlu_qPCR_R | TGGGGAAGTAGACCCTCGAC |
| *5* | *Ce*EF1_qPCR_F | AGATGCACCATGAAGCCCTC |
| *6* | *Ce*EF1_qPCR_R | GATGACCTGGGAGGTGAAGC |
| *7* | *Ce*BGlu_pRSETA_XhoI_F | GCATGACTCGAGATGGCAATTCTGAAAAGAAGTG |
| *8* | *Ce*BGlu_pRSETA_KpnI_R | GCATGAGGTACCTTAATTTGAAAATTCTGTTGCAGA |
| *9* | *Ce*BGlu_pJL-TRBO_PacI_F | GCATGATTAATTAACATGGCAATTCTGAAAAGAA |
| *10* | *Ce*BGlu_pJL-TRBO_AvrII_R | GCATGACCTAGGTTAATTTGAAAATTCTGTTGCAGA |

*encompassing partly the 5’UTR preceding the start site
