# Supplementary material for: Functional Characterization of Genes Coding for Novel β-D-Glucosidases Involved in the Initial Step of Secoiridoid Glucosides Catabolism in Centaurium erythraea Rafn
Source: Front Plant Sci. 2022 Jun 23;13:914138. doi: 10.3389/fpls.2022.914138 (PMC9260424; doi:10.3389/fpls.2022.914138)
Supplement: Supplementary file 3 [file Table_2.DOCX]

**Supplementary Table 2.** List of plant b-glucosidases and their corresponding accession numbers.

| **Abbreviation** | **Plant species** | **Protein function** | **Accession no.** |
| --- | --- | --- | --- |
| ArMY1 | *Armoracia rusticana* | Myrosinase | AAV71147.1 |
| AsCGLU1 | *Avena sativa* | Avenacosidase_1 | CAA55196.1 |
| AtBglu18 | *Arabidopsis thaliana* | Beta-D-glucopyranosyl_abscisate_beta-glucosidase | CAC19786.1 |
| AtBGLU37 | *Arabidopsis thaliana* | Myrosinase_2 | AAK28645.1 |
| AtBglu44 | *Arabidopsis thaliana* | Beta-glucosidase_44 | AEE76044.1 |
| AtBglu45 | *Arabidopsis thaliana* | Beta-glucosidase_45 | AEE33890.1 |
| AtBglu46 | *Arabidopsis thaliana* | Beta-glucosidase_46 | AEE33892.1 |
| Bjmyr1 | *Brassica juncea* | Sinigrinase | CAA11412.1 |
| Bnbgl | *Brassica napus* | Sinigrinase | CAA57913.1 |
| BnMYR | *Brassica napus* | Myrosinase | CAA42775.1 |
| BnMyr1.Bn1 | *Brassica napus* | Sinigrinase | CAA79989.2 |
| CiIpeglu1 | *Carapichea ipecacuanha* | Beta-glucosidase | BAH02544.1 |
| CeBGlu1 | *Centaurium erythraea* | Beta-glucosidase | ON060690 |
| CeBGlu2 | *Centaurium erythraea* | Beta-glucosidase | ON060691 |
| CrSTR | *Catharanthus roseus* | Strictosidine_beta-glucosidase | AAF28800.1 |
| DcBGLU1 | *Dalbergia cochinchinensis* | Dalcochinin_8-O-beta-glucoside_beta-glucosidase | AAF04007.1 |
| DlCGH | *Digitalis lanata* | Cardenolide_16-O-glucohydrolase | CAB38854.2 |
| DnBglu | *Dalbergia nigrescens* | Beta-glycosidase | AAV34606.1 |
| GmICHG | *Glycine max* | Isoflavone_conjugate-specific_beta-glucosidase | BAF34333.1 |
| GsSGD | *Gelsemium sempervirens* | strictosidine_glucosidase | AXK92564.1 |
| HsF26G | *Hellenia speciosa* | Furostanol_glycoside_26-O-beta-glucosidase | BAA11831.1 |
| HvBglu | *Hordeum vulgare* | 4-hydroxy-7-methoxy-3-oxo-34-dihydro-2H-14-benzoxazin-2-yl_glucosidebeta-D-glucosidase | AAA87339.1 |
| LeMside1 | *Solanum lycopersicum* | Beta-mannosidase | AAL37714.1 |
| LgBglu3 | *Lamium galeobdolon* | beta-glucosidase_3 | AYK02733.1 |
| LlBglu1 | *Leucaena leucocephala* | Glycosylhydrolase_1 | ABY48758.1 |
| LjBGD2 | *Lotus japonicus* | Beta-glucosidase_D2 | ACD65510.1 |
| OeGLU | *Olea europaea* | Oleuropein_beta-glucosidase | AAL93619.1 |
| OsBGLU8 | *Oryza sativa subsp. japonica* | Beta-glucosidase_8 | AAS07251.1 |
| PcCBG | *Pinus contorta* | Coniferin_beta-glucosidase | AAC69619.1 |
| PsAH1 | *Prunus serotina* | Amygdalin_hydrolase_isoform_AH_I | AAA93234.2 |
| PsBglu1 | *Prunus serotina* | Prunasin hydrolase isoform PH A | AAL07435.1 |
| PsBglu2 | *Prunus serotina* | Prunasin_hydrolase_isoform_PH_C | AAL35324.1 |
| RsRG | *Rauvolfia serpentina* | Raucaffricine-O-beta-D-glucosidase | AAF03675.1 |
| RsRMB1 | *Raphanus sativus* | Sinigrinase | BAB17226.1 |
| RsSGR1 | *Rauvolfia serpentina* | Strictosidine-O-beta-D-glucosidase | CAC83098.1 |
| RvSGD | *Rauvolfia verticillata* | Scrictosidine-beta-D-glucosidase | AFI71457.1 |
| SaMYR | *Sinapis alba* | Myrosinase_MB3 | CAA42534.1 |
| SbDhr1 | *Sorghum bicolor* | Dhurrinase | AAC49177.1 |
| SbDhr2 | *Sorghum bicolor* | Cyanogenic_beta-glucosidase_dhurrinase-2 | AAK49119.1 |
| ScBglu | *Secale cereale* | 4-hydroxy-7-methoxy-3-oxo-34-dihydro-2H-14-benzoxazin-2-yl_glucoside_beta-D-glucosidase_chloroplastic | AAG00614.1 |
| Taglu1b | *Triticum aestivum* | 4-hydroxy-7-methoxy-3-oxo-34-dihydro-2H-14-benzoxazin-2-yl_glucoside_beta-D-glucosidase_1b_chloroplastic | BAE92259.1 |
| TrBglu | *Trifolium repens* | Cyanogenic beta-glucosidase | ABV54716.1 |
| ZmGLU1 | *Zea mays* | 4-hydroxy-7-methoxy-3-oxo-34-dihydro-2H-14-benzoxazin-2-yl_glucoside_beta-D-glucosidase_1_chloroplastic | AAD09850.1 |
| ZmGLU2 | *Zea mays* | 4-hydroxy-7-methoxy-3-oxo-34-dihydro-2H-14-benzoxazin-2-yl_glucoside_beta-D-glucosidase_2_chloroplastic | AAA65946.1 |
