# Supplementary material for: Functional Characterization of Genes Coding for Novel β-D-Glucosidases Involved in the Initial Step of Secoiridoid Glucosides Catabolism in Centaurium erythraea Rafn
Source: Front Plant Sci. 2022 Jun 23;13:914138. doi: 10.3389/fpls.2022.914138 (PMC9260424; doi:10.3389/fpls.2022.914138)
Supplement: Supplementary file 4 [file Table_3.DOC]

**Supplementary Table 3.** Compounds analyzed within the present study adopting the single reaction monitoring (SRM) mode of the UHPLC/DAD/(±)HESI−MS^2^ instrument. All the compounds were analyzed in the negative ionization mode of the MS. Presented are the retention times (Rt), masses of pseudomolecular ions [M−H]^−^, masses of the two diagnostic fragments of each of the targeted compound, as well as the collision energies (cE).

| **No.** | **Compound** | **Rt (min)** | **[M−H]^−^ (*m/z*)** | **MS^2^ fragments** | **cE (eV)** |
| --- | --- | --- | --- | --- | --- |
| **1** | Swertiamarin + HCOOH | 2.41 | 419 | 119; 179 | 20 |
| **2** | Gentiopicrin + HCOOH | 2.77 | 401 | 149; 179 | 20 |
| **3** | Loganin + HCOOH | 2.86 | 435 | 127; 227 | 20 |
| **4** | Sweroside + HCOOH | 2.99 | 403 | 125; 195 | 20 |
| **5** | 1,5,9-Epideoxyloganic acid + HCOOH | 3.3 | 405 | 197; 359 | 20 |
| **6** | Vitexin | 3.34 | 431 | 269; 311 | 30 |
| **7** | Secologanin + HCOOH | 3.53 | 433 | 149; 179 | 20 |
| **8** | Isoquercitrin | 3.61 | 463 | 243; 300 | 30 |
| **9** | Apigetrin | 4.24 | 431 | 211; 268 | 30 |
