# Supplementary material for: Functional Characterization of Genes Coding for Novel β-D-Glucosidases Involved in the Initial Step of Secoiridoid Glucosides Catabolism in Centaurium erythraea Rafn
Source: Front Plant Sci. 2022 Jun 23;13:914138. doi: 10.3389/fpls.2022.914138 (PMC9260424; doi:10.3389/fpls.2022.914138)

**Supplementary Table 4.** Homology of amino acid sequences of *Ce*BGlu1 and *Ce*BGlu1, and previously characterized *β*-glucosidases from the species of Gentianales order (refer to Supplementary Table 2 for a list of plant protein sequences), expressed as amino acid sequence identity (%).


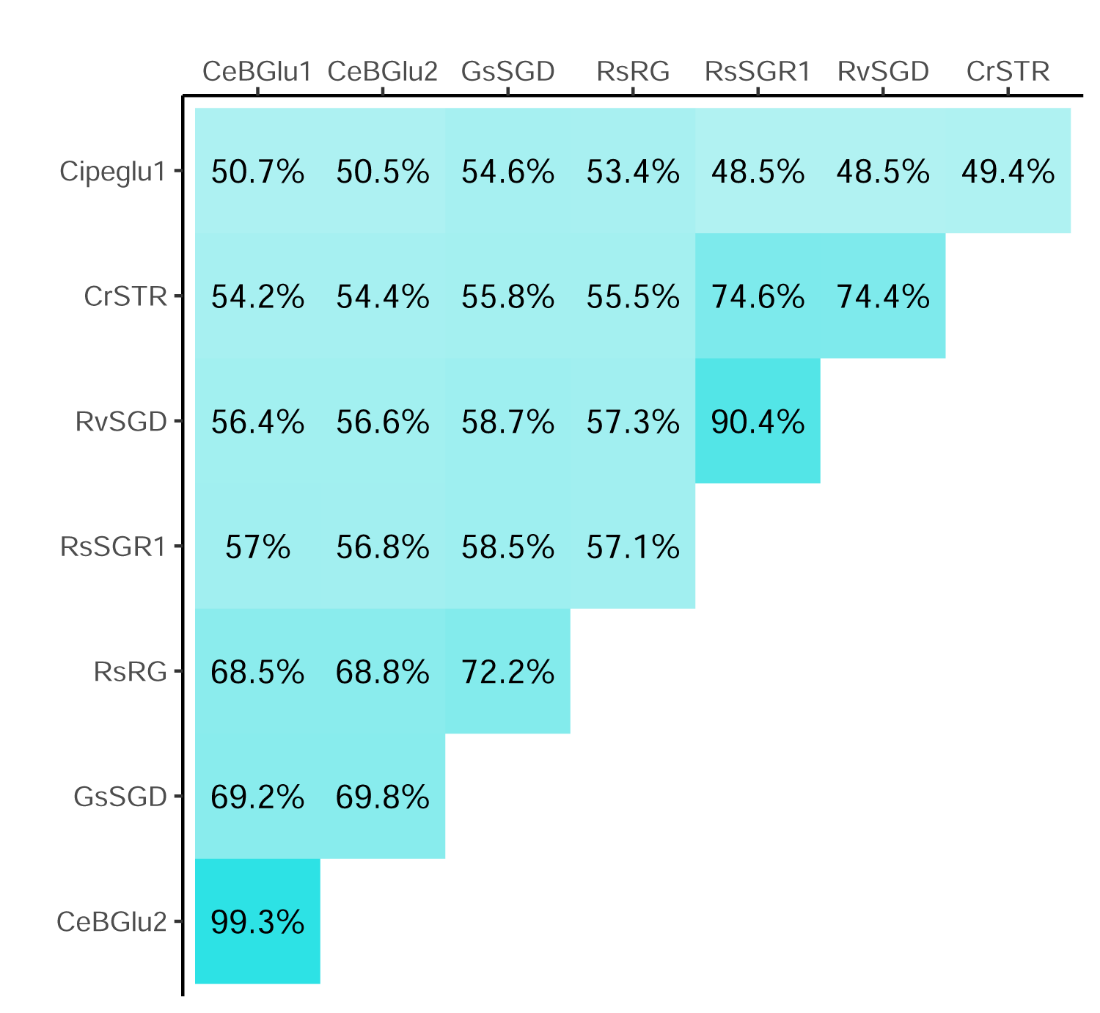

Supplement: Supplementary file 5 [file Table_4.DOCX]
