# Supplementary material for: Functional Characterization of Genes Coding for Novel β-D-Glucosidases Involved in the Initial Step of Secoiridoid Glucosides Catabolism in Centaurium erythraea Rafn
Source: Front Plant Sci. 2022 Jun 23;13:914138. doi: 10.3389/fpls.2022.914138 (PMC9260424; doi:10.3389/fpls.2022.914138)
Supplement: Supplementary file 6 [file Table_5.DOCX]

**Supplemetary Table 5**. Structural evaluation of CeβGlu AlphaFold models. Clash Score - the number of serious clashes per 1000 atoms. MolProbity Score - Combined protein quality score that reflects the crystallographic resolution at which such a quality would be expected. RMSD (root mean square deviation) was computed between aligned pairs of the backbone C-alpha atoms in superposed structures. TM-score (template modeling score) measures the topological similarity between the template and model structures (Xu and Zhang, 2010). SI% - percent sequence identity.

| **ID** | **Ramachandran favored** | **Ramachandran outliers** | **Rotamer**  **outliers** | **Clash**  **score** | **MolProbity Score** | **RMSD**  **(PDB: 3U5Y)** | **TM**  **(PDB: 3U5Y)** | **SI%**  **(PDB: 3U5Y)** |
| --- | --- | --- | --- | --- | --- | --- | --- | --- |
| *Ce*BGlu1 | 92.73% | 0.91% | 0.43% | 1.04 | 1.26 | 1.06 | 0.84 | 74 |
| *Ce*BGlu2 | 92.36% | 1.27% | 1.28% | 0.93 | 1.33 | 0.92 | 0.84 | 75 |
