# Supplementary material for: Functional Characterization of Genes Coding for Novel β-D-Glucosidases Involved in the Initial Step of Secoiridoid Glucosides Catabolism in Centaurium erythraea Rafn
Source: Front Plant Sci. 2022 Jun 23;13:914138. doi: 10.3389/fpls.2022.914138 (PMC9260424; doi:10.3389/fpls.2022.914138)
Supplement: Supplementary file 7 [file Presentation_1.PPTX]

## Slide 1
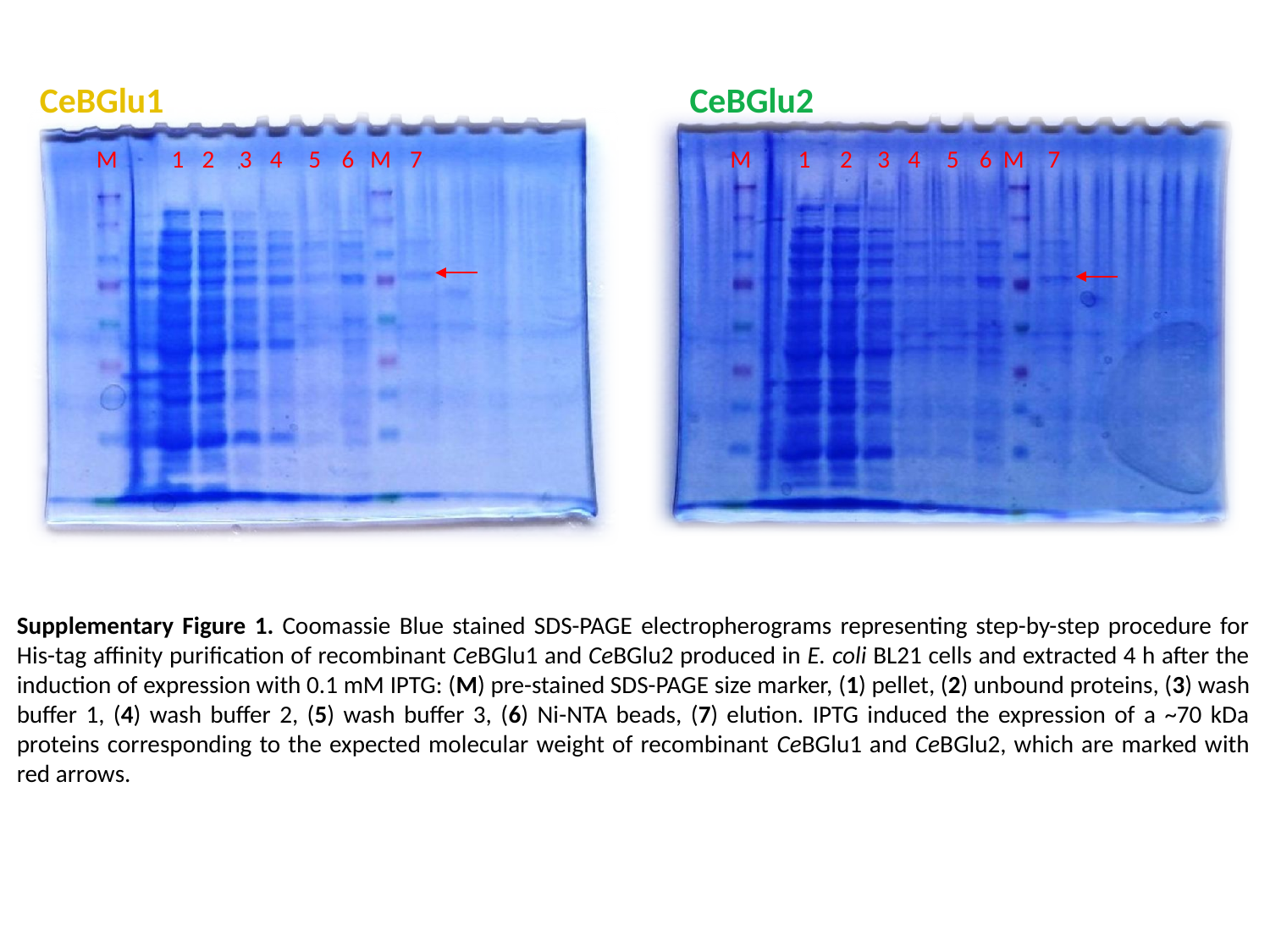

CeBGlu1
CeBGlu2
7
6
2
3
4
5
7
6
1
M
M
M
M
1
2
3
4
5
Supplementary Figure 1. Coomassie Blue stained SDS-PAGE electropherograms representing step-by-step procedure for His-tag affinity purification of recombinant CeBGlu1 and CeBGlu2 produced in E. coli BL21 cells and extracted 4 h after the induction of expression with 0.1 mM IPTG: (M) pre-stained SDS-PAGE size marker, (1) pellet, (2) unbound proteins, (3) wash buffer 1, (4) wash buffer 2, (5) wash buffer 3, (6) Ni-NTA beads, (7) elution. IPTG induced the expression of a ~70 kDa proteins corresponding to the expected molecular weight of recombinant CeBGlu1 and CeBGlu2, which are marked with red arrows.
