# Supplementary material for: Functional Characterization of Genes Coding for Novel β-D-Glucosidases Involved in the Initial Step of Secoiridoid Glucosides Catabolism in Centaurium erythraea Rafn
Source: Front Plant Sci. 2022 Jun 23;13:914138. doi: 10.3389/fpls.2022.914138 (PMC9260424; doi:10.3389/fpls.2022.914138)
Supplement: Supplementary file 8 [file Presentation_2.PPTX]

## Slide 1
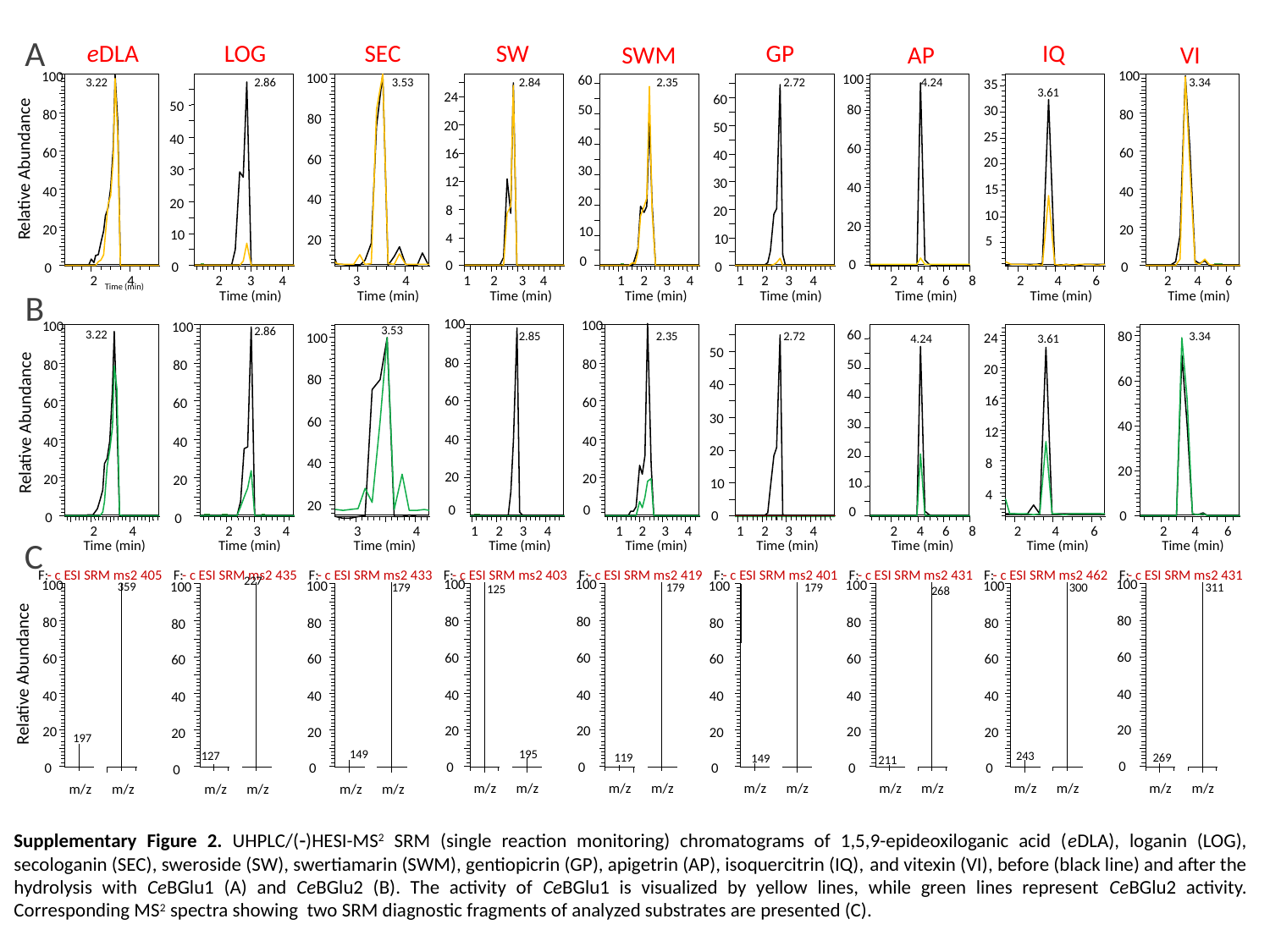

100
3.34
35
3.61
30
80
25
60
20
15
40
10
20
5
0
2
4
6
2
4
6
Time (min)
Time (min)
2
4
Time (min)
100
3.22
2.86
50
80
40
60
Relative Abundance
30
40
20
20
10
0
0
2
4
2
3
4
Time (min)
Time (min)
100
3.53
2.84
24
80
20
16
60
12
40
8
10
4
20
0
0
3
4
1
2
3
4
1
2
3
4
Time (min)
Time (min)
Time (min)
100
60
2.35
2.72
4.24
60
50
80
50
40
60
40
30
30
40
20
20
20
10
0
0
1
2
3
4
2
4
6
8
Time (min)
Time (min)
100
3.53
2.85
100
80
80
60
60
40
40
20
20
0
1
2
3
4
1
2
3
4
Time (min)
Time (min)
100
60
2.72
4.24
50
80
50
40
40
60
30
30
40
20
20
20
10
10
0
0
0
1
2
3
4
2
4
6
8
Time (min)
Time (min)
100
100
2.86
3.22
80
80
60
60
Relative Abundance
40
40
20
20
0
0
2
3
4
3
4
Time (min)
Time (min)
80
3.34
24
3.61
20
60
16
40
12
8
20
4
0
2
4
6
2
4
6
Time (min)
Time (min)
F:
- c ESI SRM ms2 405
m/z
m/z
F:
- c ESI SRM ms2 435
100
359
80
80
60
60
Relative Abundance
40
40
20
20
197
0
0
m/z
m/z
F:
- c ESI SRM ms2 433
227
100
100
80
60
40
20
127
0
m/z
m/z
F:
- c ESI SRM ms2 403
F:
- c ESI SRM ms2 419
100
179
125
80
60
40
20
149
195
0
m/z
m/z
m/z
m/z
F:
- c ESI SRM ms2 401
100
100
179
80
80
60
60
40
40
20
20
119
0
0
m/z
m/z
F:
- c ESI SRM ms2 431
100
179
80
60
40
20
149
0
m/z
m/z
F:
- c ESI SRM ms2 462
F:
- c ESI SRM ms2 431
100
300
268
80
60
40
20
243
211
0
m/z
m/z
m/z
m/z
100
311
80
60
40
20
269
0
IQ
GP
SW
SEC
LOG
eDLA
VI
AP
SWM
2.35
A
B
C
Supplementary Figure 2. UHPLC/(−)HESI-MS2 SRM (single reaction monitoring) chromatograms of 1,5,9-epideoxiloganic acid (eDLA), loganin (LOG), secologanin (SEC), sweroside (SW), swertiamarin (SWM), gentiopicrin (GP), apigetrin (AP), isoquercitrin (IQ), and vitexin (VI), before (black line) and after the hydrolysis with CeBGlu1 (A) and CeBGlu2 (B). The activity of CeBGlu1 is visualized by yellow lines, while green lines represent CeBGlu2 activity. Corresponding MS2 spectra showing two SRM diagnostic fragments of analyzed substrates are presented (C).
